# Large Language Models as Distributional Baselines for Language Tasks

**DOI:** 10.1162/OPMI.a.373

**Published:** 2026-07-18

**Authors:** Sean Trott, James Michaelov, Cameron Jones, Tyler Chang, Benjamin Bergen

**Affiliations:** Department of Psychology, Rutgers University–Newark, Newark, NJ, USA; Department of Cognitive Science, University of California, San Diego, La Jolla, CA, USA; Department of Brain and Cognitive Sciences, Massachusetts Institute of Technology, Cambridge, MA, USA; Department of Psychology, Stony Brook University, Stony Brook, NY, USA

**Keywords:** large language models, baselines, distributional semantics, predictability, behavioral experimentation, psycholinguistics

## Abstract

In order to ask questions about the mechanisms underpinning human cognition, researchers must control for properties of stimuli that could confound detected effects. In experiments involving linguistic stimuli, this includes properties like frequency, length, and neighborhood size of those stimuli, which are known to affect behavioral and neural responses. With improvements in the performance and usability of language models, it is now possible to also control for how predictable stimuli and their parts are, on the basis of the distributions of words alone: their *distributional predictability*. This coincides with a resurgence of interest in the possibility that statistical language learning may underlie a broad range of human cognitive phenomena; indeed, there are both theoretical and empirical reasons to believe that humans rely on distributional information during certain cognitive tasks. This creates a confound, whereby experimental operationalizations of psychological constructs with linguistic stimuli may not in fact be testing what they are intended to test. Thus, the central contributions of this paper are twofold: first, we articulate the conditions under which distributional predictability threatens the internal validity of an experiment; and second, we provide concrete recommendations for how to control for this potential confound. Beyond these primary contributions, we survey techniques for measuring distributional predictability, review theoretical and empirical work supporting the role of distributional statistics in human cognition, and present several case studies illustrating the range of possible outcomes—from the “distributional baselines” only marginally affecting theoretical inferences to constituting fully deflationary confounds. We also enumerate and address potential objections to this approach. This paper is primarily intended for researchers in psychology, cognitive science, and linguistics who use linguistic stimuli but have not yet incorporated distributional baselines into their work.

## INTRODUCTION

Linguistic stimuli in cognitive psychological and psycholinguistic experiments have a host of features that could confound detected effects, such as unigram frequency, bigram frequency, length, phonological neighborhood size, imageability, concreteness, and age of acquisition, among others. These features have all been empirically demonstrated to correlate with measurable outcomes of interest (e.g., behavior or brain activity), and failure to account for them can result in Type I or Type II errors or in incorrect estimates of effect sizes. To mitigate these threats, psycholinguistics researchers often control for these features—either in the design of their stimuli or in their statistical analyses—to ensure the internal validity of their research. However, one feature of stimuli is not often controlled for: the predictability of (a part of) the stimulus, given its distributional linguistic context. A large body of work demonstrates that, like frequency, length, and other features, distributional predictability is also correlated with behavioral and brain measures (Frank & Bod, 2011; Frank et al., 2015; Jones & Bergen, 2021; McDonald & Shillcock, 2003; Michaelov et al., 2022; Parviz et al., 2011; Schrimpf et al., 2021; Shain et al., 2024; Smith & Levy, 2008, 2013; Trott et al., 2023; among many others). As a consequence, like other possible confounds, distributional predictability needs to be controlled for in select cases of empirical human language processing research that tests causal claims (see below). Thankfully, recent advances in modern large language models (LLMs) have given researchers a more powerful operationalization of distributional predictability than was historically available. Probabilities of words, sequences of words, or parts of words are cheap to extract and widely accessible; they reliably predict human behavioral and brain measures, and typically do so better than past measures such as cloze (Michaelov et al., 2022; Shain et al., 2024).

The primary contributions of this paper are twofold. First, and most importantly, we articulate when and why distributional predictability represents a potential threat to the validity of an experiment (i.e., experimental designs for which there is a “distributional confound”). We provide both concrete examples (“Case Studies”), as well as a set of general principles researchers can use to determine whether they ought to control for distributional predictability (“When is distributional predictability a concern?”) and suggestions for how to do so, either in the design of experimental stimuli or in statistical analysis (“How should researchers control for distributional predictability?”). Second, we describe key decision points faced by researchers seeking to control for distributional predictability, such as the choice of model architecture or metric (“Decision Points and Best Practices”), as well as the inferential trade-offs involved in each decision. Although competing considerations and the rapidly changing technical landscape make it impossible to provide hard-and-fast “rules” for navigating this process, we do provide concrete recommendations for each decision point (see also Table 2). Beyond these two primary contributions, we briefly survey various methods for calculating distributional predictability, along with empirical and theoretical work supporting the hypothesis that distributional predictability plays a role in shaping human concepts and capacities. Finally, we conclude with a discussion of potential objections to or limitations of this approach, as well as ideas for how the field could integrate distributional baselines moving forward.

As such, this paper will be most useful for Cognitive Science, Psychology, or Linguistics researchers who use linguistic stimuli in their experiments and are investigating the role of *non-distributional* factors in cognition (e.g., innate biases, embodied or grounded experience), but may not yet be incorporating measures of distributional predictability into their design or analysis. That said, the paper also provides researchers already familiar with the research literature on distributional predictability with new, explicit criteria for assessing when distributional confounds arise and a structured discussion of methodological concerns that can serve as a useful reference.

### What is Distributional Predictability, and How is it Measured?

Research studying language processing using behavioral or neural methods often uses linguistic stimuli composed of more than one word. The distributional predictability of any such stimulus (or some part of it) is its probability, as estimated from the frequency and co-occurrence statistics of how its elements appear (e.g., in a large corpus). Often, though not always, calculating predictability will involve estimating the probability of a specific language string, conditioned on the surrounding linguistic context. For instance, consider (1):

(1)  cream and *sugar* → p(sugar ∣ cream and)

Suppose in an experiment we are interested in human processing of the final word, *sugar.* In this case, as the target word is preceded by a context including other words, the distributional probability of *sugar* is the probability of that word, given that the previous two words are *cream and*. The probability itself is typically represented as scaled to be a number bounded between 0 and 1, such that the sum of probabilities for all words in the same context (*cream and* ___) will sum to 1. Many operationalizations of predictability replace this raw probability with surprisal instead, which is equal to the negative logarithm of the probability; reasons for doing so will be discussed in more detail later on.

The probability of a word in context can be estimated in many ways—for instance, calculated from human responses (*cloze probability* is the proportion of experimental participants to fill in a gap in sentence with a given word; Taylor, 1953), generated by a syntactic parser or knowledge base, or even extracted indirectly from large language models by asking them to generate metalinguistic judgments (e.g., Dentella et al., 2023). But strictly distributional conditional probabilities are estimated based only on statistical patterns of language usage.^1^ Calculating distributional predictability thus requires the following tools: a set of vocabulary items (e.g., words, morphemes, or individual characters); a corpus (e.g., representative conversation transcripts or all of Wikipedia); and some model for estimating the probability of vocabulary items in context based on their distributional patterns in that corpus. Ultimately, the resulting system that assigns probabilities to sequences of linguistic elements (e.g., words) based only on occurrence statistics is a *statistical language model*.

### N-Gram Models

One of the oldest and simplest statistical language model approaches is the n-gram model, which estimates conditional probability based on count frequencies in a corpus (Jurafsky & Martin, 2025). For example, *p(sugar* ∣ *cream and)* could be calculated by dividing the frequency of “cream and sugar” in a large corpus by the frequency of the two-word phrase “cream and”. *N*-gram models remain useful and low-cost tools for estimating conditional probability, but suffer from several problematic limitations. First, the length of sequences under consideration (*n*, or “window size”) must be specified in advance. If the chosen window size is insufficiently large, the model will fail to make accurate predictions about stimuli, e.g., when they involve long-distance dependencies. Moreover, determining the appropriate window size involves significant trade-offs. A larger *n* can in principle facilitate more accurate predictions, particularly for longer sequences. But it can also lead to issues with data sparsity, given that the probability of observing an exact sequence decreases as the length of that sequence grows. A larger *n* also introduces computational challenges, as the number of parameters in an *n*-gram model grows (in theory) exponentially with *n* (e.g., a trigram model with 10 K vocabulary items would have approximately one trillion parameters). Various techniques have been developed to address sparsity issues (e.g., smoothing), though these techniques do not address a further, fundamental limitation of *n*-gram models, which is their lack of flexibility. *N*-gram models are used to predict specific words in specific contexts, and lack a principled mechanism for drawing generalizations across *categories* of words, e.g., “nouns” or “animate entities”. To illustrate why this is a problem, consider the following example:

(2)  I need to feed my *iguana*.

If an expression like “feed my iguana” has not occurred in an *n*-gram model’s training corpus, the probability of *p(iguana* ∣ *feed my)* would be zero (or approximately zero, with appropriate smoothing techniques). In principle, however, if an expression like “I need to feed my snake” has occurred, a language model should be able to recognize that “iguana” is grammatically and semantically similar to “snake” and thus is a plausible candidate for this slot; because *n*-gram models operate over specific strings rather than *features* reflecting the meaning or grammatical function of those strings, they lack a straightforward mechanism for identifying these similarities. At root this can be seen as a *representational* limitation, which “neural” language models are better equipped to solve.

### “Neural” Language Models

An alternative—and typically more powerful—approach involves estimating probabilities from a neural network trained on a word prediction task. These models are broadly called “neural” language models because they use computational principles from artificial neural networks that are loosely based on properties of neurons. There exist a range of computational architectures developed for the language modeling task, but a key advantage of neural models is their capacity for generalization, which is enabled by hidden layers that learn latent representations mediating the relationship between specific input words and the predicted output words. An overview of neural networks in general, or neural language models in particular, is beyond the scope of this article, but several features are relevant to distributional probability.

First, most contemporary neural language models represent vocabulary items (e.g., words) as *embeddings*: vectors of real-valued numbers occupying a lower-dimensional space than the original size of the vocabulary. For example, a vocabulary of 50 K words could be mapped to a 300-dimensional space, in which the position of each word in that space reflects similarities in their usage patterns. For example, the vectors for “iguana” and “snake” might be correlated or close in vector-space because they occur in similar grammatical and semantic contexts. This embedding space is often built using neural models, like the *word2vec* algorithm (Mikolov et al., 2013), though older techniques such as Latent Semantic Analysis (Landauer & Dumais, 1997) accomplish similar goals using related principles. The result is that vocabulary items that distribute similarly are encoded similarly in the neural language model.

Second, a neural language model is *trained* to make predictions, e.g., of upcoming words given the preceding context. This training process involves presenting examples of word sequences, as in (1) and (2), to a neural network. The neural network passes a representation of the context words (specifically, their embeddings) through its layers, which involves operating on those embeddings with a number of vectors of weights (also called “parameters”), culminating in a prediction—i.e., a probability distribution over vocabulary items. Errors made during training (e.g., assigning too high or too low a probability to a target word) are used to update the model’s parameters, eventually leading to better predictions. This training process overall serves the function of aligning the output probabilities from the model with the true probabilities in the training corpus. Importantly, because of the use of learned embeddings to represent words and the rich network structure of the model, this will often lead the model to generalize in a data-driven way beyond the examples it has seen.

Finally, neural language models have demonstrated remarkable advances in recent years, stemming from multiple factors, including: larger corpora for training data, increased computational resources, larger models (i.e., more parameters), and new model architectures (like transformers) that exploit the benefits of scale more effectively. Due to the vastness of their training data and parameter count, these models are often called large language models (LLMs). LLMs show improved performance at nearly all benchmarks over their predecessors, scaling with number of parameters and training corpus size. (Note, however, that scale does not always accompany improved psychometric predictive power; see “Decision Points and Best Practices”.) This includes improvements in their core performance metric, their prediction accuracy. Most prominent among these at present is the Transformer architecture, which makes use of the self-attention mechanism (Vaswani et al., 2017) for identifying relevant relationships between items in the context. Like *n*-gram models, Transformers use a fixed context window, but these context windows are often much larger, consisting of thousands to hundreds of thousands of tokens, where the term *token* is used to refer to an item in the language model’s vocabulary^2^. This, in combination with their neural network architecture makes them considerably more powerful, underlying well-known systems like the GPT (“generative pre-trained transformer”) family of models. This means that all things being equal, newer, larger models trained on more data likely better reflect the true probabilities of vocabulary items in a corpus (see, e.g., Brown et al., 2020; Kaplan et al., 2020).

## WHY DOES DISTRIBUTIONAL PREDICTABILITY MATTER?

There are both theoretical and empirical reasons for taking distributional predictability seriously in the design and analysis of psycholinguistic and other language-based psychology experiments. Drawing on recent reviews of the topic (Boleda, 2020; Connell, 2019; Lenci, 2008; Louwerse, 2018; Lupyan & Winter, 2018), we provide a review of relevant work below.

The *distributional hypothesis* positions the distributional statistics of language as a source of knowledge about linguistic structure and meaning (Firth, 1957; Harris, 1954; Lupyan & Winter, 2018) and even the world (Lewis et al., 2019). Lenci (2008, p. 3) describes the distributional hypothesis as follows: “The degree of semantic similarity between two linguistic expressions A and B is a function of the similarity of the linguistic contexts in which A and B appear”. Put another way: “You shall know a word by the company it keeps” (Firth, 1957). According to these accounts, the distributional patterns of linguistic expressions like words—such as which words they co-occur with—acts as a cue to their grammatical and semantic function, suggesting that a statistical learner with access to sufficient data should be able to *infer* these properties with some degree of reliability. For example, a distributional learner might infer that “dog” and “cat” share a number of syntactic and semantic features (both nouns, both animate entities, both common pets); moreover, that same learner might bootstrap their existing knowledge of “dog” and “cat” to a novel word “gub” with an analogous distributional profile.

Indeed, a number of contemporary theoretical “hybrid” accounts of semantic knowledge posit some role for distributional statistics (Louwerse, 2018; Lupyan & Winter, 2018), often operating alongside grounded experience in the world (Andrews et al., 2009). These accounts typically distinguish between the conditions under which distributional information is likely to be recruited. One such distinction concerns the kind of word, with some accounts positing that language statistics are particularly important for shaping our representations of *abstract* words (Lupyan & Winter, 2018). Other accounts emphasize the depth of processing required, suggesting that language statistics support “good-enough” representations sufficient for shallow or rapid tasks, perhaps serving as a kind of “linguistic shortcut” (Connell, 2019). Similarly, Louwerse (2018) argues that representations of word meaning can be derived *both* from perceptual experiences and from the linguistic contexts in which the target word operates—and that comprehenders may rely on the latter to “bootstrap” meaning from a limited set of grounded referents. Notably, while these accounts primarily focus on the representation of word meanings, some accounts of “higher-level” cognitive capacities (such as world knowledge or social reasoning) also emphasize the role of linguistic exposure (Dove, 2020; Lupyan et al., 2026). Crucially, each of these theories suggests that distributional statistics are a theoretically plausible *path* to knowledge. Thus, distributional predictability—the likelihood of a word or phrase in some linguistic context, as derived from its distributional profile—should be considered as a competing theoretical alternative in any account emphasizing the role of non-linguistic or innate sources of human knowledge or behavior.

One form of empirical evidence for this hypothesis comes from the observation that metrics derived from statistical language models, such as the probability of a word in context (i.e., distributional predictability), exhibit sensitivity to experimental manipulations such as grammaticality (Futrell et al., 2019; Hu et al., 2024; Linzen et al., 2016), semantic or physical plausibility (Jones & Bergen, 2024b; Kauf et al., 2023; Michaelov & Bergen, 2020), and even pragmatic or communicative context (Hu et al., 2023; Trott et al., 2023). These models have also advanced considerably in their performance on Natural Language Processing (NLP) benchmarks, including tasks designed to assess their semantic and pragmatic understanding (Wang et al., 2018).

Crucially, metrics like distributional predictability are also predictive of various kinds of *human* behavior, e.g., in online language processing tasks, as well as offline judgments about language stimuli. There is now a large body of research establishing an empirical relationship between distributional predictability and reading time (e.g., Frank & Bod, 2011; Frank et al., 2013; McDonald & Shillcock, 2003; Shain, 2019; Shain et al., 2024; Smith & Levy, 2008, 2013; Staub, 2011); typically, reading times are longer for less predictable words or word sequences, suggesting that processing difficulty could in part reflect distributional predictability. Note, however, that the picture is complicated. There is also evidence that distributional predictability *fails* to reliably account for variance in human reading time in a range of cases—notably, this includes syntactically complex constructions, where surprisal tends to *underestimate* processing difficulty (Huang et al., 2024); this suggests that the mechanisms underlying processing of complex constructions may involve factors that distributional predictability fails to capture. Moreover, even in cases where surprisal does correlate with reading time, it does not always emerge as the dominant or only predictor (Levy et al., 2013). Finally, as we note elsewhere (see “Best Practices”), there is some debate about whether language models that are better at predicting words are *also* better at predicting human reading time (Oh et al., 2024; Wilcox et al., 2023).

Distributional predictability is also correlated with measures of brain activity, such as the N400 component of the EEG signal (Aurnhammer & Frank, 2019; Frank et al., 2015; Merkx & Frank, 2021; Michaelov, Arnett, & Bergen, 2024; Michaelov, Bardolph, et al., 2024; Michaelov et al., 2022; Szewczyk & Federmeier 2022) and the fMRI BOLD signal (Hosseini et al., 2024; Schrimpf et al., 2021). Finally, other distributionally-derived metrics—such as the cosine distance between vector representations—can also be used in regression frameworks to predict human behavior, including judgments about word similarity (Haber & Poesio, 2024; Li & Joanisse, 2021; McDonald & Ramscar, 2001; Nair et al., 2020; Rivière et al., 2025; Rivière & Trott, 2025; Trott & Bergen, 2021), reading time (Trott & Bergen, 2023), and the N400 component (Michaelov, Bardolph, et al., 2024; Van Petten, 2014; Vinaya et al., 2024, 2025). Of course, these metrics also diverge from human behavior in important ways (Ettinger, 2020; Linzen & Leonard, 2018; Michaelov et al., 2025) and often fail to account for theoretically relevant variance in human behavior (Jones & Bergen, 2024b; Trott & Bergen, 2023). Yet the fact that they correlate with meaningful outcome measures (e.g., reading time, N400 amplitude) lends empirical support to the notion that distributional predictability—and distributional statistics more generally—may play a part in human language processing, and in cases where they are a plausible source of tested behavior, that they need to be controlled for in studies of other causal variables that use linguistic stimuli.

A final source of evidence comes from experimental studies using an artificial word learning paradigm, which ask whether human subjects can learn properties of novel words from their distributional statistics. These studies suggest that distributional learning is facilitated by the coherence of new concepts with existing lexical knowledge (Ouyang et al., 2017); and further, that words acquired through distributional statistics may nonetheless “inherit” the sensorimotor associations of known words with similar distributional profiles (Günther et al., 2018). Together with the evidence reviewed above, these studies are consistent with “hybrid theories”, in which distributional statistics play *some role* in semantic knowledge and in language processing alongside additional, non-linguistic mechanisms (Andrews et al., 2009; Louwerse, 2018; Louwerse & Jeuniaux, 2010).

## WHEN IS DISTRIBUTIONAL PREDICTABILITY A CONCERN FOR INTERNAL VALIDITY?

As discussed above, measures of distributional predictability frequently co-vary with common dependent measures of human language processing (e.g., reading time, N400 amplitude). Thus, while there is no evidence that distributional predictability offers a complete account of human language processing or cognition (see also “Objections”), our argument is that it can pose a candidate threat to internal validity for studies meeting two specific criteria.

1. The study measures behavioral or neural responses to—or generation of—linguistic stimuli, where stimuli are modified in systematic ways across experimental conditions to reflect an independent variable of interest, or where differences in generated language are the dependent measure.
2. The study is investigating a claim about the role of a factor *other* than distribution-based probability, including innate mechanisms or representations acquired through embodied, grounded, or situated world experience.

The first criterion concerns the nature of stimuli used to *operationalize* the theoretical construct of interest. Intuitively, a study using imagistic stimuli is not vulnerable to confounds relating to the distributional predictability of words or words sequences; of course, images might be vulnerable to a range of other potential confounds, potentially even including those involving distributional learning of visual input, though this is not our primary focus here (see “Grounded Models” in the “Best Practices” section). Many psycholinguistic studies, however, do operationalize the construct of interest (e.g., grammaticality, plausibility, pragmatic felicitousness, etc.) using sets of linguistic stimuli. In many (though not all) cases, these stimuli are constructed as “minimal pairs” across conditions, such that the only intended difference between stimuli across conditions reflects the experimental variable of interest. For instance, an experiment manipulating grammaticality might alter only whether the verb agrees with the number of the subject (e.g., “the keys to the cabinet are” vs. “the keys to the cabinet is”). Similarly, an experiment investigating the role of plausibility in language processing might manipulate only the plausibility of the described event while holding other variables constant (e.g., “When the steel ball smashed into the glass vase, the vase broke” vs. “When the steel ball smashed into the glass vase, the ball broke”). In each case, distributional predictability could in principle vary across experimental conditions, e.g., grammatical or plausible sentences might be assigned a higher probability by a language model than ungrammatical or implausible ones (Hu et al., 2026). Any such difference would represent a potential *distributional confound*.

The second criterion concerns the theoretical claim being investigated. Distributional predictability poses a confound specifically when a study is investigating a factor whose causal origins are presumed to be something *other* than distributional statistics. This could include innate grammatical knowledge, embodied or sensorimotor simulation, world knowledge derived from non-linguistic experience, pragmatic reasoning, Theory of Mind, and more. In contrast, distributional predictability is (obviously) *not* a confound for studies investigating the role of distributional statistics specifically; indeed, such a study might instead be vulnerable to alternative confounds that a researcher would need to control for. Moreover, in some studies, researchers might be interested in establishing a cognitive phenomenon without making strong claims about its underlying mechanism or causal origin. In these cases, the distributional baseline approach could in principle be used to ask whether distributional language statistics provide a plausible mechanism for the phenomena, though it would not represent a confound to the alternative explanation presented by researchers.

Both criteria are important for determining whether distributional predictability is a concern for a given study. A study using imagistic stimuli to investigate sensorimotor simulation would not be directly vulnerable to confounds relating to the distribution of words or word sequences, nor would a study using linguistic stimuli to investigate distributional learning from language statistics. If both criteria are met, however, distributional predictability does represent a *candidate* threat to the study’s internal validity. Note that it does not follow from both criteria holding that distributional predictability necessarily “explains away” the experimental variable of interest; that is an *empirical question* contingent on the application of the distributional baselines method (see “How should researchers control for distributional predictability?”). Instead, these criteria can be viewed as prerequisites for distributional predictability being a candidate theoretical confound. Again, to draw another analogy to word frequency: researchers control for frequency in their design or analysis not because it is guaranteed to “explain away” the effect of some variable of interest, but because there are both theoretical and empirical reasons to believe that it might.

The two criteria mentioned above are thus theoretical in nature, as opposed to empirical. That is, they reflect properties of a study design (criterion 1) and the causal variables of interest in accounting for measured behavior on the study (criterion 2). As noted above, our claim is not that internal validity is *necessarily* compromised in such a scenario but that it could be, and that this potential threat merits the consideration of distributional predictability as a potential alternative causal explanation. Naturally, the threat posed to internal validity is strengthened if two additional *empirical* criteria concerning a specific body of experimental data are met:

3.  A measure of distributional predictability (e.g., surprisal) reliably covaries with the experimental condition of interest.
4.  The measure of distributional predictability also reliably covaries with the dependent measure (e.g., response time).

If these four criteria are met, distributional predictability clearly represents a threat to internal validity. In such a scenario, the relevant outcome measure (e.g., reading time) *could* be driven not by the causal factor intended in the study’s design, but instead, as demonstrated in the previous section, by variance in the distributional predictability of linguistic strings across experimental conditions. Failure to account for distributional predictability—in the design of stimuli, analysis of linguistic outputs, or in the statistical analysis—could in principle lead to either Type I (false positive) or Type II (false negative) errors with respect to inferential interpretations of the experimental hypothesis under investigation. Below, we lay out the theoretical case for how a failure to account for distributional predictability could generate both kinds of error. We then discuss how researchers might go about controlling for distributional predictability, and describe the results of several empirical studies doing so.

### Distributional Predictability and Signal Detection

Empirical studies can make at least two kinds of error: they might incorrectly reject the null hypothesis (a “false positive”), or they might incorrectly fail to reject the null hypothesis (a “false negative”). Both errors are costly to the scientific enterprise, and in research involving linguistic stimuli specifically, both could plausibly be driven by a failure to account for distributional predictability.

The case for possible *false positives* is relatively straightforward to establish. Suppose a team of researchers is investigating Theory X, which posits a role for some non-linguistic mechanism in language processing. The researchers design a study with carefully crafted linguistic stimuli in two conditions (A and B); according to Theory X, processing should be more difficult in Condition B than Condition A. The researchers then measure reading time (RT) as a proxy for processing difficulty, and find that average RT is indeed significantly longer in Condition B than Condition A. Unbeknownst to the researchers, however, the stimuli also vary in their distributional predictability; further, distributional predictability is the *true* generative mechanism responsible for variance in RT. In this scenario, distributional predictability is a confound with respect to the theoretical variable of interest, and failure to account for it could lead to a false positive.

This scenario is abstract, so here is a more concrete example: suppose Theory X claims that “language understanding depends on physical world experience”, and conditions A and B contain linguistic descriptions of events varying in their physical plausibility (such that A > B), as in Jones and Bergen (2024b). If humans read sentences in Condition A faster than Condition B, that might be counted as support for Theory X. Yet it is possible that distributional predictability—derived from corpora containing descriptions of events—might co-vary with physical plausibility, and further, that human responses reflect knowledge gain from linguistic distributions rather than sensorimotor experience. In this case, failing to account for distributional predictability would leave the researchers open to a Type I error—thus, the authors should control for predictability in their analysis.

A *false negative* is also in principle possible, though *a priori* seems less likely than false positives. That said, consider the case where distributional predictability exhibits the *inverse* relationship with the outcome variable, compared with the experimental manipulation of interest. If both the experimental manipulation and distributional predictability influence the outcome variable, their effects could “cancel out”, leading to a failure to detect the effect of the experimental manipulation on the outcome variable if distributional predictability is not accounted for. For example, Rohde et al. (2021) found that in some circumstances, more informative sentences describing situation atypical events (e.g., “she was chopping the carrots with a shovel”) can be processed more quickly than less informative ones (e.g., “she was chopping the carrots with a knife”). Because informativity and distributional predictability will often be inversely correlated, failing to control for the effects of distributional predictability on comprehension could mask informativity effects.

Although we have focused here on a simple experimental design (a single condition with two levels), the same concerns apply to more complex designs, including interaction effects or correlations between two or more continuous variables. Additionally, and importantly, the correlations (or anticorrelations) between the distributional predictability and theoretical predictions need not be complete or even particularly strong for Type I or II errors to occur—they need only be strong enough to shift the variance explained by experimental conditions enough to cross significance thresholds, given the inferential statistics scheme used for analysis. Finally, beyond Type I or II errors that lead to incorrectly rejecting or not rejecting the null hypothesis, distributional predictability may shift measured effect sizes so that effects are estimated to be weaker or stronger than the true effects. As we discuss below, there are effective strategies to reduce the likelihood of either type of error.

### How Should Researchers Control for Distributional Predictability?

Similar to how research using language addresses other potential confounds, there are two approaches to addressing possible effects of distributional predictability, through design or through statistical analysis. These approaches are not, of course, unique to distributional predictability, nor are they novel to our proposal; the suggestion we flesh out here is that researchers simply also apply widely-used methods to potential “distributional confounds”. In the current section, we discuss these approaches, then review several case studies from published literature using what we call the “distributional baselines” approach. Interested readers can find example R code demonstrating this method at the following GitHub repository: https://github.com/seantrott/baselines_demo/. For researchers interested in computing surprisal directly, we recommend the *minicons* library (Misra, 2022), which provides a straightforward interface for extracting token-level and sequence-level probabilities from various language models.

First, researchers can control for distributional predictability in the *design* of their stimuli. This is analogous to “balancing” stimuli according to other known confounds, such as frequency, concreteness, or age of acquisition. In this case, researchers explicitly design stimuli—often through an iterative process—such that the average distributional predictability is “matched”, i.e., not significantly different, across experimental conditions. One relatively early example of this approach applied to distributional statistics is Glenberg and Robertson (2000), which matched stimuli across conditions for their average cosine distance to a target word (see also Case Study 1 below). However, perfect matching is not always practical or feasible, particularly as modern LLMs appear to exhibit more sophisticated linguistic behaviors or even semantic knowledge, making it more difficult to design “adversarial” stimuli for which LLM-derived distributional predictability would show *no* effect. That is, we anticipate it may often be impossible to completely eliminate differences in distributional predictability across experimental conditions while retaining a large enough number of quality stimuli for the human research in question (see also the Case Studies below). Parenthetically, we note that while selecting adversarial stimuli that are completely matched for surprisal may be possible in some cases, it also carries with it the risk of inadvertently introducing *selection effects*, such that the resulting stimuli are not necessarily as representative of the construct of interest as some counterfactual stimulus set that has not undergone this matching process—possibly reducing, eliminating, or artificially inflating a human effect as well. Unfortunately, the question of whether a given stimulus set is representative of the construct of interest is not one we can resolve here, and it is also not unique to the challenge of accounting for distributional predictability.

Consequently, while researchers will often be well served by attempting to control for distributional predictability in stimulus design, we advocate more generally for a second approach: partialling out its effect statistically. This partialling out approach is only effective for reducing Type I errors, as Type II errors may persist in select cases and can be better addressed by matching predictability across conditions as described above. But for Type I errors, researchers can adjust for distributional predictability in their statistical analyses by including it as a covariate. We suggest following a *model selection approach*, which has become more common as an analysis tool in cognitive psychology and psycholinguistics research.

One standard approach to model selection is the use of nested model comparisons. Here, a “full” regression model is compared to a reduced model that omits only a specific independent variable of interest. A common comparison method is a log-likelihood ratio test (LRT), which compares the likelihood of the full model to the reduced model. If the data are significantly more likely under the full model than under the reduced model, this suggests that the variable of interest adds significant explanatory power, i.e., it accounts for significant variance over and above the other variables included in the reduced model.

When this approach is deployed to deal with distributional predictability, the full model includes both relevant predictors, e.g., the experimental Condition and a measure of distributional predictability (such as Surprisal, discussed below). An example of such a model specification, which also includes by-participant random slopes for each factor, is given in (a) below. The reduced model, given in (b) below, omits only the experimental condition, but preserves all other model structure from (a).

(a)  Full model: RT ~ Condition + Surprisal + (1 + Condition + Surprisal ∣ Participant)
(b)  Reduced model: RT ~ Surprisal + (1 + Condition + Surprisal ∣ Participant)

Using statistical software like R (R Core Team, 2021) and specific packages like *lme4* (Bates et al., 2015), researchers can construct both models and conduct a likelihood-ratio test. If the full model explains significantly more variance than the reduced model, it provides evidence that the outcome variable is not fully explained by distributional predictability, and thus that distributional predictability is not sufficient to capture effects of the causal factor manipulated across conditions and tested in the experiment.

Note that researchers will also face the same range of analysis decisions as for any statistical analysis, and which are not discussed at length here, such as which random effects to include (Barr et al., 2013), whether and how to center variables before entering them into a regression (Gelman & Hill, 2007), whether and which additional covariates (such as word frequency) to account for (Goodkind & Bicknell, 2021), whether and how to control for spillover effects, i.e., the influence of preceding words’ surprisal on the processing of the current word (van Schijndel & Linzen, 2021), and more. Here, we refer readers to papers more directly targeted at the question of appropriate statistical procedures (Barr et al., 2013; Gelman & Hill, 2007; Winter, 2019). Moreover, a likelihood ratio test is only one method for model comparison and selection. Researchers could use other methods as well, such as Akaike Information Criterion (Burnham et al., 2011; for examples see, e.g., Michaelov, Arnett, & Bergen, 2024; Michaelov, Bardolph, et al., 2024; Michaelov & Bergen, 2024; Vinaya et al., 2024), prediction error using cross-validation, or Bayesian analyses (Nicenboim et al., 2025; Vasishth et al., 2018) (e.g., those specifying prior distributions for parameter estimates, and/or reporting measures of fit such as Bayes Factors). An exhaustive comparison of the advantages and disadvantages of different approaches is beyond the scope of the current manuscript, though we note that researchers might even employ multiple methods in the service of determining how robust a given theoretical conclusion is to exact design decisions (Steegen et al., 2016; see also “Best Practices”).

In each case, the goal is to establish whether the experimental condition contributes explanatory power to a statistical model over and above a variable reflecting distributional predictability. Note that these approaches are distinct from merely asking whether distributional predictability is *sensitive* to the experimental contrast, which is already commonly done in psycholinguistics and computational linguistics research. The “distributional baselines” question is not whether distributional statistics produce *some* sensitivity to the manipulation, but whether and to what extent they account for the effect of that manipulation on human behavior. The full statistical model created in this approach can also be used, outside of the model selection paradigm, when the objective is to estimate the effect size of the experimental variable—*independent* of distributional predictability. Researchers can (again) include distributional predictability as a covariate in a full model and assess the coefficient for the variable of interest.

### Case Studies

There is now a growing body of research deploying this “distributional baselines” method (DeLong et al., 2023; Jones & Bergen, 2024a; Jones et al., 2022, 2024a; Michaelov et al., 2021, 2022; Michaelov, Bardolph, et al., 2024; Trott & Bergen, 2023; Trott et al., 2023). We focus on several case studies in particular; taken together, these empirical studies illustrate several points. First, while they are not an exhaustive survey of potential applications of the baselines method, they do showcase a range of studies and experimental variables that are *potentially* vulnerable to a distributional confound, e.g., from work on physical world knowledge to mental state reasoning. Second, despite this variance in the theoretical construct of interest, each study investigates the target construct by measuring human responses to linguistic stimuli (as is common in psycholinguistics). These properties correspond to the first two criteria described in the previous section (“When is distributional predictability a concern for internal validity?”). Third, the case studies illustrate how statistically accounting for distributional predictability can work in practice, thus serving as a guide to interested researchers.

Crucially, the case studies also serve to highlight key *empirical results* concerning distributional predictability. For instance, they show that distributional predictability does often, in fact, covary both with an experimental variable of interest (criterion #3) and with a dependent measure of human behavior on a task (criterion #4). Concretely, distributional predictability is *correlated* with human behavior across a range of dependent measures and experimental manipulations. This correlation does not, of course, entail that distributional predictability is the causal mechanism responsible for behavior on the task, nor does it even entail that it better accounts for human behavior than the experimental variable of interest. Indeed, as the case studies demonstrate (see also Figure 1), there are a range of potential empirical outcomes that can arise from applying the baselines method, ranging from distributional predictability failing to account for any condition-relevant variance in human behavior to fully accounting for condition-relevant variance in human behavior. These results are summarized in Table 1, and select results are reproduced in Figure 2.

**Figure 1.**
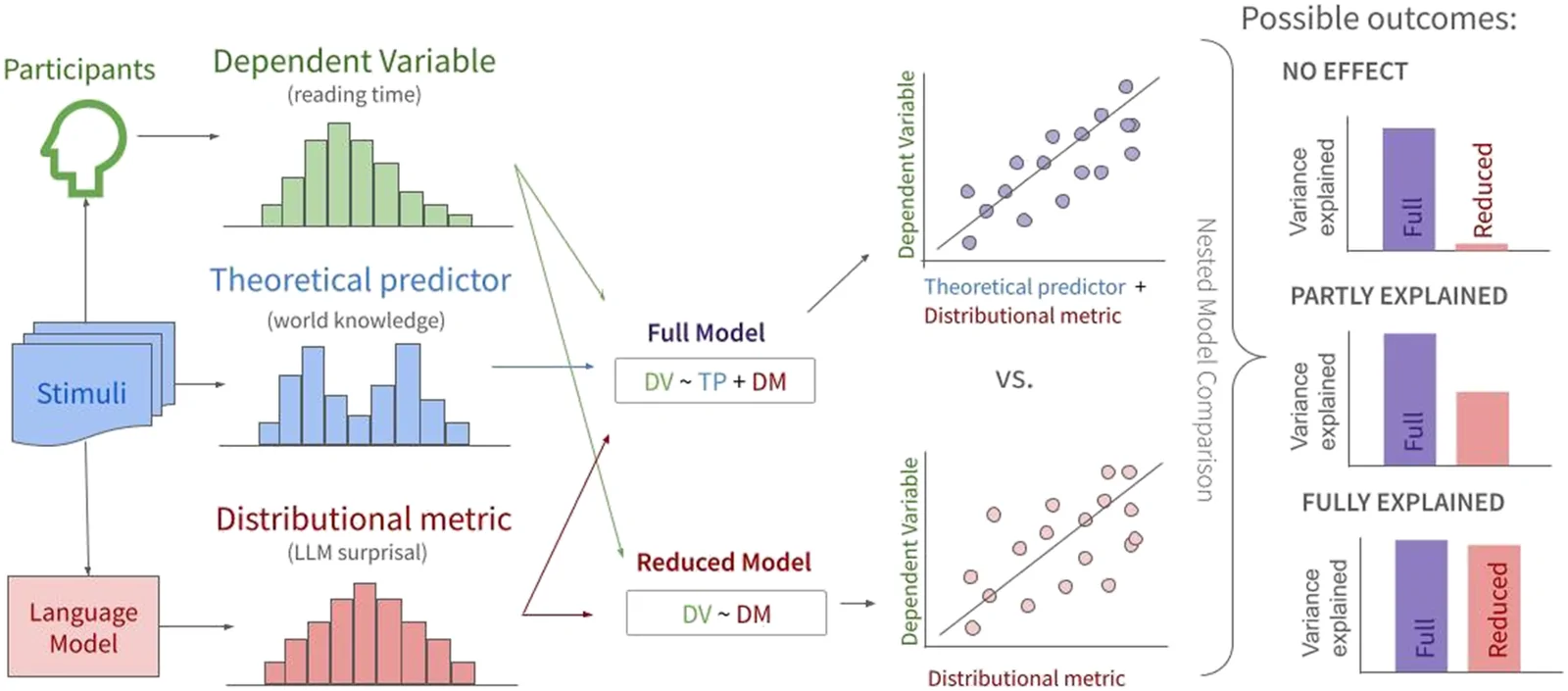
A visual representation of a typical baselines procedure. Stimuli are designed that vary according to some theoretical predictor which constitutes the experimental contrast. The same stimuli are presented to both human participants and a language model, producing a dependent variable of human processing and a measure of distributional predictability respectively. We compare a full model (with both predictors) to a reduced model (using just the distributional metric to predict the DV). This comparison might indicate that the distributional metric explains (A) no variance in human behavior, (B) some, but not all of the variance explained by the full model, or (C) all of the variance that the theoretical predictor can explain. Note that the red bar in the “Partly explained” panel also corresponds to the “Reduced” model, as in the other panels; the label is omitted for space.

**Table 1.** Summary of the case studies described below. Possible outcomes include “None” (i.e., surprisal does not account for any condition-relevant variance, or possibly any variance at all, in human behavior), “Partial” (surprisal accounts for some but not all condition-relevant variance in human behavior), and “Full” (surprisal fully accounts for the effect of the experimental variable on human behavior).

**Case Study**	**Experimental Manipulation**	**Measure of Distributional Predictability**	**Outcome**
#1	Affordedness	Surprisal	Partial
#2	True vs. false beliefs	Log Odds (Surprisal Diff.)	Partial
#3	Cloze probability	Surprisal	Full
#3	Contextual relatedness	Surprisal	Full
#3	Semantic plausibility	Surprisal	Full
#4	Physical plausibility (Study 1)	Log Odds	Partial
#4	Physical plausibility (Study 2)	Surprisal	None
#5	Same vs. different sense	Cosine Distance	Partial

**Figure 2.**
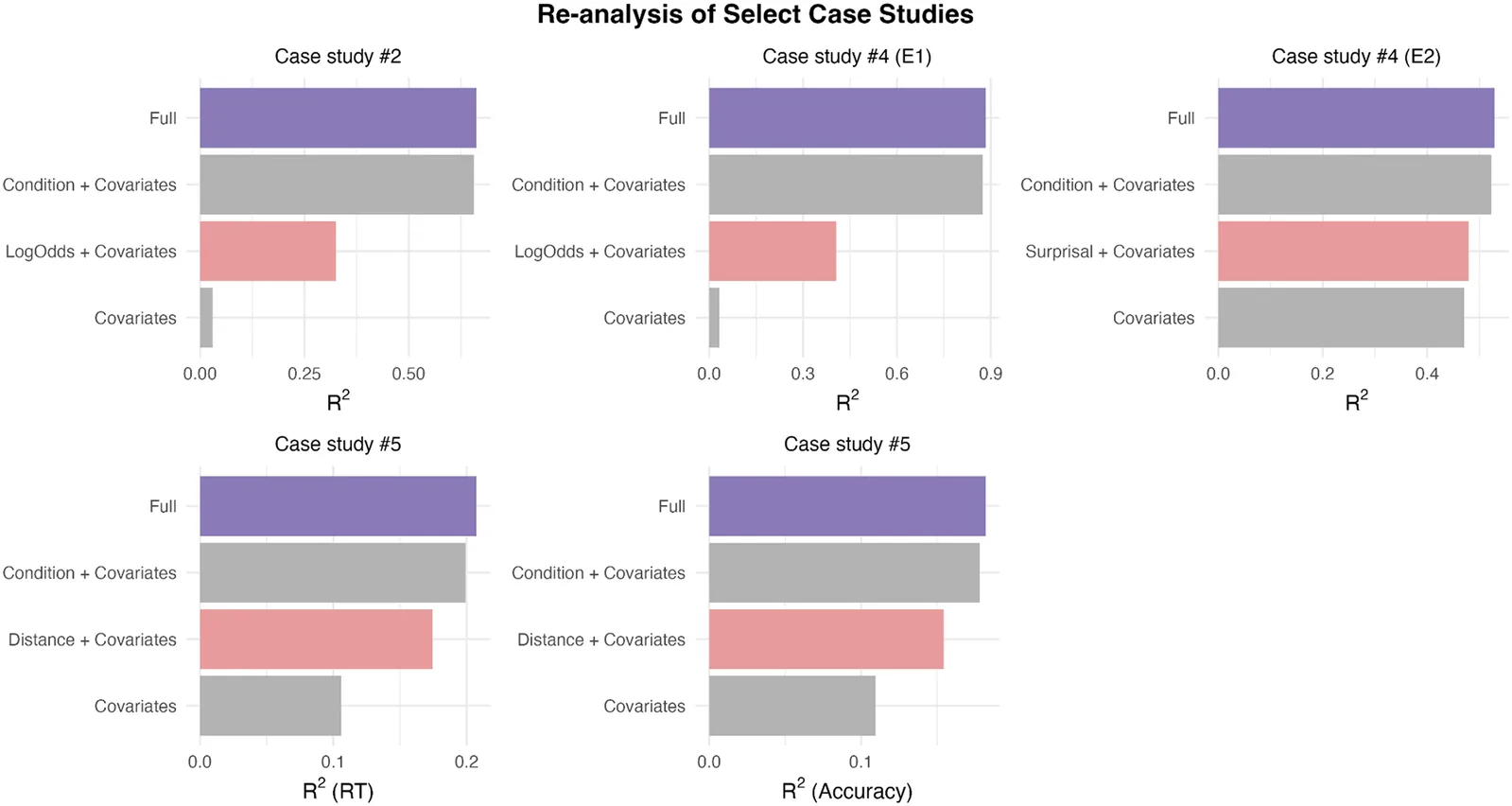
Results of re-analyzing by-item aggregate data from select case studies, involving manipulations of belief states (Case Study #2; Trott et al., 2023), physical plausibility (Case Study #4; Jones & Bergen, 2024b), and the presence or absence of a sense boundary between two usage contexts for an ambiguous word (Case Study #5; Trott & Bergen, 2023).

### Case Study #1: Distributional Predictability and Object affordances

A key question in the study of language processing is the extent to which physical world experience is necessary for understanding the concepts expressed in language—and to what extent those concepts can be learned from language itself. Glenberg and Robertson (2000) investigated this question by asking about the ability of humans to identify unusual *affordances* of objects—i.e., the actions that some organism could perform with that object. Some affordances are canonical and likely learnable directly from language (e.g., that a towel can be used for drying), but others might plausibly rely on direct world experience (e.g., that a shirt can be used for drying). The authors found that humans were able to identify novel affordances, but that a measure derived from distributional statistics was not. However, this study relied on much older distributional models, and it also did not directly compare distributional predictability to human behavior using the baselines approach we outlined above.

More recently, Jones et al. (2022) addressed both limitations in a replication and extension. First, Jones et al. (2022) found that surprisal from certain state-of-the-art LLMs (e.g., GPT-3) was, in fact, correlated with the key experimental manipulation—thus posing a threat to internal validity. Second, Jones et al. (2022) asked whether LLM surprisal explained away the effect of the experimental manipulation on *human* behavior, and found that it did not. While the manipulation explained around a third of the variance in human sensibility judgements, GPT-3 surprisal explained just 13%. This case study thus reflects a scenario in which distributional predictability was able to *partially* (but not *fully*) explain away the effect of the experimental manipulation on human behavior.

### Case Study #2: Distributional Predictability and Theory of Mind

Theory of Mind (ToM) is the ability to attribute mental states to other social agents. There is considerable debate about the origins of human ToM—specifically, whether ToM is an innate, evolutionary adaptation (Bedny et al., 2009) or whether it is constructed from experience (Hughes & Ensor, 2005). One “experiential” theory posits a strong role for language experience in particular, suggesting that exposure to mental state verbs (Brown et al., 1996) and complex syntax (Hale & Tager-Flusberg, 2003) could scaffold the ability to reason about mental states. This makes distributional predictability useful not only as a “control” variable but as a potential explanatory mechanism: if distributional predictability explains human behavior on a ToM task, it would suggest that ToM could in principle result from language exposure alone; but if the experimental manipulation explains additional variance in human behavior, it would suggest that human-level ToM depends on some additional, non-linguistic mechanism—either non-linguistic experience or an innate ToM module.

One study (Trott et al., 2023) tested this hypothesis by obtaining measures of distributional predictability from LLMs for a language-based false belief task (false belief tasks being an important operationalization of Theory of Mind). Distributional predictability was measured as the relative log probability (log-odds) assigned by a language model (GPT-3) to a character looking for an object in the start (incorrect) vs. end (correct) location. They found that log-odds was sensitive to the key experimental manipulation: namely, GPT-3 assigned higher probability to a character looking in the start location when the character didn’t know the object had been moved. That is, GPT-3 was *sensitive* to a character’s belief states, providing empirical support for the concern that distributional predictability might represent a potential threat to internal validity.

The authors also ran a human cloze task with the same scenarios, in which human participants were instructed to complete a sentence based on the passage they had just read (e.g., “John looks for the book in the ___”). They then used the nested model comparison described earlier to ask whether the experimental condition explained variance in human behavior (whether they wrote in the start vs. end location) above and beyond GPT-3 log-odds. Specifically, they compared a full model with fixed effects of both knowledge state and log-odds to a reduced model omitting only the effect of knowledge state. They found that the full model displayed significantly better fit, i.e., the effect of knowledge state on human behavior was not explained away by distributional predictability. As with Case Study #1, this outcome reflects a *partial explanation* of the human effect by distributional predictability. More recent work (e.g., Jones et al, 2024; Trott et al., 2026) has extended this approach to a broader range of ToM tasks and additional LLMs, with similar results.

### Case Study #3: Distributional Predictability and the N400

The N400 (Kutas & Hillyard, 1980) is a neural index of language processing; specifically, it is a negative-going deflection in the event-related brain potential that peaks roughly 400ms after the presentation of a meaningful stimulus. In language, the N400 response is sensitive to the extent to which the word is frequent, contextually predictable, semantically congruent with its context, or any of a range of other factors; though the question of precisely which factors shape this reduction in N400 amplitude, and the neurocognitive mechanisms involved, are still widely debated (Federmeier, 2022; Kuperberg et al., 2020; Kutas & Federmeier, 2011; Rabs et al., 2022; Van Petten & Luka, 2012).

One explanation of the N400 is that it reflects the difficulty of integrating a given word into its preceding context, with more difficult integration leading to larger (more negative) amplitudes (Nieuwland et al., 2020; van Berkum et al., 1999). Alternatively, under preactivation accounts, the N400 reflects new activation (or retrieval) of a given word and its meaning elicited by encountering the word; and instead differences in N400 amplitude index the extent to which the word or its meaning was already activated by the preceding context before the word was encountered. This can either be explained as arising due to prediction (e.g., Kuperberg et al., 2020) or due to contextual relatedness, i.e., the semantic relations between the context and the word (e.g., Federmeier, 2022; Rabs et al., 2022). It is worth noting that many accounts of the N400 involve combinations of these (e.g., Federmeier, 2022; Nieuwland et al., 2020; Rabs et al., 2022; Szewczyk & Federmeier, 2022).

While predictability has historically been operationalized as cloze probability, more recent work has shown that language model surprisal can predict N400 amplitude well (Frank et al., 2015; Michaelov et al., 2022; Michaelov, Bardolph, et al., 2024). This suggests the need for a distributional baseline—could the brain simply be predicting upcoming words based on the statistics of language, and is this sufficient to explain the N400?

In one study, Michaelov et al. (2022) test whether language model surprisal (and thus, prediction based on the statistics of language) is sufficient to explain the effect of predictability on the N400, finding that surprisal from 3 language models (GPT-3, ALBERT, and RoBERTa) show a closer fit to the N400 data, and explain variance in N400 amplitude not explained by cloze probability, while cloze probability explains no additional variance above and beyond the surprisal calculated from any of these models; suggesting that the distributional baseline may be sufficient to account for the effect of predictability on the N400. Thus, the relationship between cloze probability and the N400 amplitude can be *fully accounted for* by surprisal.

Michaelov, Bardolph, et al. (2024) take this one step further, investigating whether the distributional baseline of GPT-3 can explain not only the predictability effects, but also the effect of contextual relatedness (hypothesized to directly impact N400 amplitude in contextual relatedness accounts; see, e.g., Federmeier, 2022; Szewczyk & Federmeier, 2022) and plausibility (hypothesized to directly impact N400 amplitude based on the integration account; see, e.g., Nieuwland et al., 2020). Michaelov, Bardolph, et al. (2024) find GPT-3 surprisal explains all the variance explained by cloze probability, contextual relatedness, and plausibility (but not vice-versa); suggesting that the prediction account may be able to account for these implicitly rather than them requiring additional neurocognitive architectures; and furthermore, that prediction based on the statistics of language may be sufficient for this. Thus, while language model predictions do not necessarily account for all reported N400 effects or explain all the theoretically meaningful variance in the data (see, e.g., Krieger et al., 2025; Michaelov, Bardolph, et al., 2024; Michaelov & Bergen, 2020; Szewczyk & Federmeier, 2022), the distributional baseline can serve an important deflationary role. Specifically, in both of the cases outlined here, a measure of distributional predictability (surprisal) *fully accounted* for some alternative variable of interest (i.e., cloze probability, semantic plausibility, or contextual relatedness).

### Case Study #4: The Role of World Knowledge in Pronoun Resolution

Jones and Bergen (2021, 2024b) used the distributional baselines approach to measure the extent to which purely linguistic knowledge could account for the role of world knowledge in pronoun comprehension. In sentences like (3), the pronoun *it* is ambiguous, in that it could refer to either *the rock* or *the vase*.

(3)  When the rock fell on the vase, it broke.

Several theories suggest that comprehenders will use their knowledge about the world to resolve the ambiguous pronoun to the most plausible referent: here, *the vase* (Graesser et al., 1994; Hobbs, 1979; Sanford & Garrod, 1998). It is often assumed that this world knowledge would have to be nonlinguistic—either based on pre-experiential core knowledge or sensorimotor experience of the physical world. However, distributional predictability provides an alternative explanation: comprehenders could prefer *the vase* because the wordform *vase* may be more closely associated with the word *break* in the statistics of corpora that comprehenders have been exposed to.

To test these hypotheses, Jones and Bergen (2024b) developed 30 sentences like (3), and measured their world knowledge bias by asking a group of participants which of the interpretations was more plausible. A second group of participants read the sentences and answered a comprehension question which implicitly probed how they resolved the pronoun (e.g., “What broke?”). The authors also presented the same sentences to GPT-3 text-davinci-002: a 175bn closed-weights autoregressive LLM developed by OpenAI (Brown et al., 2020). Based on OpenAI’s API documentation at the time, text-davinci-002 had been pre-trained on more than 200 billion words, and then fine-tuned on examples of instruction-following, but not fine-tuned using reinforcement learning from human feedback.

In order to estimate the probability that the model assigned to each potential interpretation of the pronoun, the authors replaced the pronoun with each of the possible antecedents and found the sum log probability that the model assigned to the tokens which followed the pronoun:

(4)  a. *log*(*p*(broke. ∣ When the rock fell on the vase, the rock))
  b. *log*(*p*(broke. ∣ When the rock fell on the vase, the vase))

This technique had been found to be effective for knowledge-driven pronoun resolution in other settings, such as the Winograd Schema Challenge (Kocijan et al., 2023; Radford et al., 2019).

The authors used nested model comparison to measure the marginal explanatory power of the world knowledge norms. First, they fit a base binomial mixed effects model that used GPT-3 surprisal to predict participant's responses. This base model fit significantly better than a null model (*χ*^2^(1) = 20.1, *p* < 0.001; marginal *R*_2_ = 0.121) that included the syntactic bias of the sentence, suggesting that GPT-3 predictions were significantly correlated with participants’ pronoun interpretations. However, including world knowledge as a predictor significantly improved fit over a model that included GPT-3 surprisal (*χ*^2^(1) = 50.6, *p* < 0.001; marginal *R*_2_ = 0.555). This suggests that world knowledge was explaining additional variance that was not being captured by the language model.

In a second experiment, Jones and Bergen (2024b) used a self-paced reading paradigm to investigate participants’ spontaneous interpretation during reading (i.e., without being prompted by a comprehension question). Participants read the same sentences as in Experiment 1, but followed by a continuation sentence that contradicted one of the possible pronoun interpretations (e.g., “the [rock/vase] was still intact”). The authors were interested in whether participants would slow down more when they read a continuation that contradicted the more plausible interpretation: suggesting that they had already spontaneously committed to the interpretation during reading.

In this study, the authors measured the sum surprisal of each of the self-paced reading regions, conditioned on the passage up to that region. They fit regression models predicting log reading time of the critical region (containing the contradiction) and 3 spillover regions. A base model included the syntactic bias of the sentence, the participants’ reading speed before the critical sentence, and the average reading time for an unambiguous version of the same sentence. A second model that included GPT-3 surprisal for the predicted region (as well as the 3 preceding regions, to measure spillover effects) was no better at predicting reading times than the base model. However, including world knowledge as a predictor significantly improved the fit of the model predicting reading time in the region immediately following the contradictory information. In the full model, there was no significant effect of any of the GPT-3 surprisal predictors.

Collectively, the results suggest that distributional statistics contain some information about how comprehenders use plausibility to resolve pronouns. However, this information cannot explain the extent to which direct plausibility judgements predict pronoun resolution. In turn this is consistent with theories that suggest some resource beyond language itself is necessary for resolving these ambiguities—whether reasoning, core knowledge, or sensorimotor simulation.

### Case Study #5: Are Word Meanings Categorical or Continuous?

Many words have multiple meanings, i.e., they are *ambiguous*. A long-standing question in psycholinguistics is how humans represent these multiple meanings. One view is that different meanings (e.g., “dog *bark*” vs. “tree *bark*”) are represented as distinct “entries” in a kind of mental dictionary, thus emphasizing the categorical, discrete nature of word senses. An alternative view—rooted in recurrent neural network (RNN) models—characterizes meanings as “trajectories” through a continuous state-space (Elman, 2009), eschewing the notion of discrete senses altogether. The topology of this meaning space could in principle be shaped by multiple factors, but one particularly relevant factor is the *distributional usage patterns* of words. Until recently, operationalizing this hypothesis was challenging due to limitations of distributionally-derived word representations.

Recent work (Trott & Bergen, 2023) attempted to adjudicate between these views using contextualized word representations from BERT (a transformer language model). Specifically, Trott and Bergen (2023) constructed a number of minimal sentence pairs containing the same ambiguous word. In some pairs, both contexts selected the same sense (e.g., “He liked the marinated lamb” vs. “He liked the roasted lamb”); in other pairs, the contexts selected different senses (e.g., “He liked the marinated lamb” vs. “He liked the friendly lamb”). The authors then calculated the cosine distance between contextualized embeddings for the target word (e.g., *lamb*) for each sentence pair: as expected, Different Sense contexts produced larger cosine distances than Same Sense contexts.

Crucially, the authors then ran a human experiment in which participants performed a series of primed sensibility judgments. Participants read a prime sentence containing an ambiguous word in one context (e.g., “He liked the marinated lamb”) followed by a target sentence containing that same ambiguous word (e.g., “He liked the friendly lamb”), which corresponded to either the Same Sense or a Different Sense. Trott and Bergen (2023) measured both accuracy (whether participants correctly identified the target as sensible) and response time (how quickly participants responded correctly). The authors then asked about the relative explanatory power of the discrete sense boundary variable (Same vs. Different Sense) as well as Cosine Distance derived from contextualized BERT representations. For both dependent variables (accuracy and response time), a *full* statistical model including both predictors—as well as a number of lexical covariates, such as the frequency and concreteness of the target word—was compared to a reduced model omitting only the experimental variable of interest. The authors also compared the full model to a reduced model omitting only Cosine Distance to determine whether Cosine Distance explained additional variance in human behavior. For both dependent variables (accuracy and response time), Same vs. Different Sense explained *additional variance* above and beyond Cosine Distance. At the same time, Cosine Distance accounted for unique variance beyond Same vs. Different Sense, and beyond the lexical covariates of interest.

Together, these results are consistent with the interpretation that distributional statistics *partially account* for the effect of sense boundaries on word processing, but do not *fully account* for this effect. Moreover, this pattern of results has been reproduced using other metrics such as surprisal (Trott & Bergen, 2023), other dependent variables like relatedness (Trott & Bergen, 2021), and in other languages such as Spanish (Rivière et al., 2025).

### Summary and Re-Analysis

The case studies described above show that distributional predictability can and does account for meaningful variance in human behavior across a range of tasks. In many cases, this includes condition-relevant variance, i.e., distributional predictability *partially* or even *fully* (as in Case Study #3) accounts for the relationship between some other experimental variable and the dependent measure. They also highlight the range of possible *empirical outcomes* that arise from applying the baselines method (see Table 1). In some cases, distributional predictability may fully eliminate the effect of interest, while in others, it may explain no variance in human behavior at all. This is an empirical question: only by actually accounting for distributional predictability in the context of a given experimental study can researchers determine whether an experimental manipulation contributes explanatory power above and beyond what is available from distributional statistics alone—and thus draw causal inferences about the theoretical constructs responsible for observed behavior.

As a further concrete demonstration that distributional predictability can explain variance in human behavior on experimental tasks, and to harmonize these results with the presentation of the baselines method in Figure 1, we re-analyzed select case studies using a common measure of variance explained (*R*^2^) and present the results in Figure 2 below. For each re-analyzed study, we report several statistics: 1) the *R*^2^ of a “null” model omitting both the experimental variable of interest and the measure of distributional predictability, but with other relevant covariates; 2) the *R*^2^ of a model with only distributional predictability (and covariates other than the experimental variable); 3) the *R*^2^ of a model with only the experimental variable of interest (and covariates other than distributional predictability); and 4) the *R*^2^ of a model with all variables. We focused on Case Studies #2, #4, and #5, as both had publicly available data on GitHub. For ease of interpretation, we report *R*^2^ values from ordinary least squares (OLS) models rather than from mixed-effect models; we verified in each case that the qualitative pattern of results (i.e., whether distributional predictability fully accounted for condition-relevant variance) was consistent with an analysis of the full dataset using likelihood ratio tests and mixed-effects models.

#### Re-analysis of Case Study #2.

In Trott et al. (2023), participants read a short passage involving a manipulation of a character’s belief states, then indicated where they thought that character would look for an object (i.e., either the start or end location of an object). The effect of the experimental manipulation (True vs. False Belief) was compared to the effect of Log Odds (i.e., the probability assigned to the start vs. end location by a language model). We re-analyzed the data using the by-item mean response rates (i.e., the proportion of participants who predicted a character would look in the start location), fitting models with the experimental manipulation (True vs. False Belief), the Log Odds for that item, as well as several covariates included in the original study (e.g., which location was mentioned first). As shown in Figure 2, both distributional predictability and the experimental manipulation explained more variance in human behavior than the covariates alone (~3%): the model with covariates plus Log Odds explained approximately 33% of by-item variance, while the model with all variables (including the experimental manipulation) explained approximately 66% of the variance. A model containing only the experimental manipulation (along with covariates) explained 65.5% of the variance, indicating that distributional predictability added very little information *beyond* the effect of experimental manipulation, whereas the experimental manipulation alone explained almost twice as much variance as distributional predictability alone. This is consistent with the original interpretation from Trott et al. (2023), namely that distributional predictability *partially* accounted for the effect of the experimental manipulation, but did not *fully* account for the effect; at the same time, distributional predictability did meaningfully explain variance in human behavior, above and beyond other covariates.

#### Re-analysis of Case Study #4.

Jones and Bergen (2024b) asked whether distributional predictability was sensitive to the physical plausibility of an event (e.g., a rock breaking a vase) in the context of a pronoun resolution task. Pronoun resolution was measured either in terms of an answer to a comprehension question (in Experiment 1) or reading time on a continuation that was either consistent or inconsistent with the physically plausible interpretation (in Experiment 2). For each study, we reproduced the key *R*^2^ values from each of the statistical models used to predict human behavior: a model with only lexical or syntactic covariates (e.g., the syntactic bias of the sentence), a model including either distributional predictability or the experimental manipulation (i.e., physical plausibility), and a model including all predictors. In Experiment 1, distributional predictability accounted for more variance in human behavior (40.7%) than the covariates (3.2%); however, physical plausibility alone accounted for more than twice the variance of predictability (87.4%). In Experiment 2, distributional predictability barely represented an improvement in explanatory power (49.7%) over the model with only covariates (47.1%); the model with physical plausibility represented a modest improvement over both (52.5%). Thus, Experiment 1 illustrates a situation in which distributional predictability *partially accounts* for human behavior but does not *fully account* for the effect of condition; Experiment 2 illustrates a situation in which distributional predictability provides *little to no explanatory power* above and beyond other covariates.

#### Re-analysis of Case Study #5.

Trott and Bergen (2023) asked whether human behavior (as measured by response time and accuracy) in a primed sentence plausibility task varied as a function of whether the prime and target sentence belonged to the same meaning or different meanings, as well as the distributional distance between those contexts. Using publicly available data from Trott and Bergen (2023), we conceptually reproduced the original analyses on by-item mean response times and by-item mean accuracy rates. For both dependent variables, we fit OLS models with the experimental manipulation (Same vs. Different Sense), distributional distance (cosine distance using BERT embeddings), and the lexical covariates included in the original analysis (concreteness of the target ambiguous word, log frequency of the target ambiguous word, number of words in the target sentence, and the part of speech of the target ambiguous word). As depicted in Figure 2, distributional distance accounted for a larger proportion of variance in both by-item average response time (17.4%) and by-item average accuracy (15.5%) than the covariates alone (11% and 10.6%, respectively): that is, it explained significant variance in human behavior. At the same time, a full model including the manipulation of sense boundaries explained even more variance in both response time (20.7%) and accuracy (18.2%). This is consistent with the interpretation in Trott and Bergen (2023) that distributional predictability partially accounted for the effect of sense boundaries, but did not fully account for it. Moreover, we reproduced this qualitative pattern of results when analyzing trial-level data with mixed-effects models and comparing those models with a likelihood ratio test.

## DECISION POINTS AND BEST PRACTICES

Using statistical language models as *distributional baselines* requires a number of methodological decisions. Many come with trade-offs. In this section, we review common decision points involved in the distributional baselines approach, and present recommendations about how to navigate these trade-offs. Note that what constitutes “best practices” is subject to change (e.g., as the landscape of available models and tools continues to evolve). Thus, where possible, we have attempted to emphasize the inferential consequences of different decision points, rather than making narrow recommendations about specific models or tools, as we hope that these suggestions will retain broader relevance and assist researchers in making decisions most appropriate to their theoretical and methodological goals. These decision points and recommendations (as well as suggestions about when, if ever, to deviate from the recommendations) are summarized in Table 2 at the end of this section.

**Table 2.** Summary of design considerations and default recommendations, as well as suggestions about when (if ever) to deviate from default recommendation.

**Design Consideration**	**Default Recommendation**	**When to Deviate**
Architecture	Auto-regressive transformer	Use bidirectional models when participants have full context
Model scale	Start with largest open model, then iterate with smaller models if largest model explains away effect	Start with smaller models when the research question explicitly concerns developmental plausibility
Training regime	“Base model” trained on standard language modeling objective	N/A: we do not recommend deviating from “base” models when testing the sufficiency of distributional statistics
Prompting	Use surprisal of words or word sequences directly; incorporate a *prefix* (e.g., instructions or examples) when necessary for the experimental design.	Prompt model for explicit responses when measuring the probability of the target string does not correspond to the construct of interest; note that the surprisal of response options can still be used as a criterion even in this situations.
Degree of Openness	Prioritize open-weight and open-data models	If required to use closed-source models for performance reasons, also test open-source models.
Data contamination	Create new stimuli	If using existing stimuli, audit training data or use contamination detection methods
Grounded models	Avoid for tests of distributional sufficiency	Use for hypotheses about multimodal language processing (beyond scope of current paper)
Metric	Use surprisal (negative log-probability)	Use other metrics (e.g., cosine similarity between embeddings) when required by task structure (e.g., similarity judgments between items); consider other transformations when model fit is poor

### Which Model is an Appropriate Baseline?

As Ong (2024) notes, there is a wide range of statistical language models on offer. These models vary in a number of methodologically relevant parameters. Which model is most appropriate to use as a baseline? Here, we consider the trade-offs involved in selecting an LLM as a distributional baseline, and offer recommendations for how to navigate these trade-offs.

Note that in select cases, other statistical language modeling approaches (e.g., *n*-gram models) may be more appropriate, for instance when operating in lower-resource language contexts; in the sections below, we focus primarily on statistical language models using neural networks.

#### Model Architecture.

The currently dominant language model architecture is the *transformer* (Vaswani et al., 2017), which makes use of the self-attention mechanism. Older architectures, such as recurrent neural networks (Elman, 1990), generally exhibit a poorer empirical fit to human behavioral data than transformers (Michaelov et al., 2022). Thus, although the design of transformers was not motivated by cognitive plausibility per se, their relatively superior “psychometric predictive power” (PPP–Wilcox et al., 2023) makes them suitable distributional baselines. This recommendation might change if or when other architectures become more performant, for example in the case of modern recurrent architectures or selective state-space models (for discussion, see Michaelov, Arnett, & Bergen, 2024).

Within the category of transformers, the primary architectural distinction is between *auto-regressive* and *bidirectional* models. Auto-regressive models (sometimes called unidirectional or “causal” models) assign probabilities to tokens or sequences of tokens conditioned only on the preceding context; for example, in English, a unidirectional model would typically be trained to predict an upcoming token based on tokens on the left side of the target token. Bidirectional models condition their output on the tokens on both sides of the target token. To illustrate the distinction, a left-to-right unidirectional model would assign the same probability to “spoon” in both 5a) and 5b), because the left context is the same in each case—whereas a bidirectional model would assign different probabilities in each case, as “spoon” is not equally likely when considering the following context.

(5)  a. She used a spoon to stir the tea.
  b. She used a spoon to slice the onion.

For most language tasks (e.g., self-paced reading), auto-regressive models such as GPT-2 will be more appropriate, under the assumption that human comprehenders do not generally have access to words that occur after the target token—although this may not be strictly true due to foveal and parafoveal reading (Rayner & Bertera, 1979); and it is also worth noting that it is possible to use bidirectional models to calculate the probability of words based only on their previous context (Kauf & Ivanova, 2023; Michaelov et al., 2022; Misra et al., 2020; Samuel, 2024). In some cases, then, such as non-final cloze tasks, where the participant considers the whole text before making a response, a bidirectional model (such as BERT or RoBERTa) is likely to be more appropriate.

#### *Recommendation*.

Thus, for most online language processing tasks (e.g., self-paced reading, EEG), in which a participant responds to linguistic items incrementally without the use of forward context, we recommend the use of auto-regressive models. For tasks where the participant responds to words (or phrases, etc.) using the full context, we suggest using a bidirectional model. Note that one other consideration here is that auto-regressive models have generally seen more recent development and improvements.

##### Model Scale.

LLMs vary considerably in their *scale*, in terms of the amount of training data (i.e., number of training tokens), the size of the model itself (i.e., the number of parameters), and even the context window (the number of tokens a model can attend to in any given forward pass). Empirically, increases in model scale are often correlated with improvements in model performance on the language modeling task (i.e., lower perplexity), as well as other common NLP benchmarks (Kaplan et al., 2020). Does this entail that researchers should look to the biggest available LLM as a distributional baseline?

One consideration is empirical: if larger models exhibit better PPP, then it is reasonable to use a larger model as a “best guess” or “upper bound” for how much information can be learned from distributional statistics alone—whereas using a smaller model trained on less data might disadvantage the distributional hypothesis, potentially leading to false positives. Model scale is often positively correlated with PPP, though there are some exceptions: for instance, some researchers (Kuribayashi et al., 2021; Michaelov & Bergen, 2026; Oh et al., 2022; Oh & Schuler, 2023) have found that surprisal estimates from larger models with lower perplexity actually exhibit worse empirical fit to certain dependent measures (specifically, reading time). One possible explanation for this result is that larger models are actually better at predicting rare words, which produces a worse fit to human RT for those words (Oh et al., 2024). However, other work (Wilcox et al., 2023) has found positive correlations between a language model’s ability to accurately predict the next word and its fit to human reading time; it is possible this discrepancy arises from differences in the number of tokens each model was trained on. Further, larger models with lower perplexity have been shown to produce better fits to other dependent measures, such as BOLD activation (Schrimpf et al., 2021) and N400 amplitude (Michaelov, Arnett, & Bergen, 2024). More recent work (Rivière et al., 2025) is also consistent with the finding that parameter count is positively correlated with PPP when predicting relatedness judgments.

A second consideration is theoretical: even if larger models with lower perplexity exhibit better PPP, these models are generally trained on orders of magnitude more words than the average human encounters in their lifetime (Hart & Risley, 1992; Warstadt et al., 2025). As others have pointed out (Wilcox et al., 2023), models might thus *overestimate* the informativity of distributional statistics, leading to an unfair advantage for the distributional hypothesis—and potentially leading to false negatives with respect to the alternative hypothesis in question. This issue has led to interest in building and evaluating language models trained on developmentally realistic amounts of data (Hosseini et al., 2024; Warstadt et al., 2025), as well as using data that more closely resembles the nature of linguistic input children routinely encounter (see “Training Regime” below). It is possible that the relevant inferences might change if or when future models can “do more with less”.

It is worth distinguishing between two reasons sometimes given for *not* defaulting to the largest model with the lowest perplexity. Theoretically, the best, largest models might not be operationalizations of distributional learning; here, the threat is that distributional statistics could explain more than they “should” with respect to human cognition (see also “Are LLMs too good?”). On the other hand, empirically, powerful models’ ability to predict infrequent words may *decrease* their PPP. Here, the concern is that our estimate of the information provided by distributional statistics will be too low, rather than too high—biasing the results towards false positives for the alternative hypothesis.

##### *Recommendation*.

We recommend an iterative approach, considering the potential costs of both false positives and false negatives. First, in the interest of avoiding false positives (i.e., with respect to the alternative hypothesis), we suggest that researchers start with the *best available* (and ideally open-source; see “Model Source”) model for an upper bound of what is in principle achievable through distributional statistics. Here, “best available” could be interpreted in several ways. One natural interpretation appeals to model scale, given that larger models often (though not always, i.e., as with naturalistic reading time) have better PPP. A different interpretation appeals to perplexity, under the assumption that models that better predict upcoming words will also have better PPP (though again, as discussed above, this is not always true). Of course, researchers could also select models on the basis of PPP for a closely related task, though this may not always be tractable. Our default suggestion is to use scale as a proxy for model quality when no PPP estimate is available, though we are not firmly committed to any particular operationalization of “model quality”; we note, however, that researchers should be careful not to select models on the basis of their PPP on the target task itself.

Regardless of how the best available model is selected: if this model fails to explain away the experimental variable in question, this provides a strong case *against* the sufficiency of distributional statistics. If this model *does* explain away the experimental variable, we suggest that researchers replicate this finding with smaller, more developmentally plausible models to assess the robustness of the result to specific modeling choices. This iterative approach is similar to what Steegen et al. (2016) term a “multiverse analysis”, which helps establish the robustness of a given scientific inference. As with measures of unigram frequency (which depend on the size and source of the corpus), no single estimate will be perfect or conclusive.

Along these lines, more recent work (Trott et al., 2026) used a suite of open-weight models ranging in their number of parameters and amount of training data to provide a *distribution* of PPP estimates, which could then be compared to model-level properties (e.g., size). Measuring the PPP of a distribution of models avoids some of the challenges involved in identifying which *single* model to use: instead of reporting a single PPP estimate, researchers could report properties of the distribution (e.g., its median and range). Of course, this approach cannot entirely sidestep the fundamental questions here: for instance, if a single model fully accounts for condition-relevant variance but other models account for none, what should a researcher conclude? Our view is somewhat nuanced here: technically, only a single example is required to serve as a sufficiency proof (see also “How generalizable is this finding?”); at the same time, one might be (intuitively) more convinced by a demonstration across a range of models varying in size and training protocol. This kind of inductive reasoning is especially important for claims about the insufficiency of distributional statistics, which cannot rely on the deductive logic of an existence proof in the same way: here, demonstrating that a range of LLMs fail to fully account for condition-relevant variance strengthens the claim that distributional statistics are an insufficient mechanism. Of course, in all cases, researchers should be cautious about generalizing the results of a single model to the broader class of possible distributional learners, and should report which properties of a model are correlated with its PPP (e.g., its size). This is another reason to support an iterative approach that involves testing multiple models.

##### Training Regime.

Another crucial factor that determines language model behavior is the training regime or “curriculum”. This includes the *kind* of training data (e.g., written text vs. conversation transcripts), as well as specific training *protocols* or procedures (e.g., the standard language modeling task vs. reinforcement learning from human feedback).

The training data for most language models consists primarily of written text from the Internet (Bender et al., 2021; Grieve et al., 2025), which is not representative of all language varieties that constitute the broader language or languages in question (Grieve et al., 2025). Just as with the *amount* of training data, this raises the question of whether these language models are fair operationalizations of the distributional hypothesis, given that human children generally do not learn language by reading Wikipedia. In our view, the field would benefit from more targeted, developmentally plausible training curricula, and we are excited about efforts to do so (Warstadt et al., 2025). At present it is not possible to maximize both the developmental plausibility of training data and also model performance, but we recommend adopting the practice of using models with the most plausible training data possible, all other things being equal; see Misra and Mahowald (2024) for an example of studying the acquisition of grammatical constructions in language models using a “controlled rearing” method.

In terms of training protocols, the standard language modeling task is well suited to operationalize the distributional hypothesis, as it involves training a model to make better predictions about upcoming (or masked) tokens on the basis of the immediate linguistic context. However, many state-of-the-art language models—particularly more recent models designed for chat responses—are subject to additional training procedures, such as reinforcement learning from human feedback (RLHF) (Ouyang et al., 2022), from AI feedback (RLAIF) (Lee et al., 2023), or from verifiable rewards (RLVR) (Shao et al., 2024). All of these involve updating a model’s parameters on the basis of explicit feedback, e.g., human (or AI) rankings of possible model outputs. Thus, all introduce information into the training signal that is arguably absent from the next-token prediction task. For example, when ranking model outputs, humans likely draw on explicit world knowledge or cultural norms and their judgments shape the model’s subsequent output. As a result, the model is no longer merely learning to predict words from their distributional profile. Therefore, while reinforcement-tuned models are interesting objects of study in their own right—and may be useful for other purposes, such as data annotation (Martínez et al., 2024; Trott, 2024; Ziems et al., 2024) or studying how humans interact with models (Jones & Bergen, 2024a)—they are less suitable operationalizations of the distributional hypothesis. (A similar argument applies to fine-tuning models on human-annotated data, such as parse trees, semantic role labels, or relatedness judgments. It also extends to models trained with instruction-tuning, given that this procedure (again) introduces additional information into the training procedure: specifically, curated instruction/response pairs demonstrating which kinds of responses are “appropriate” for which kinds of instructions or questions.)

##### *Recommendation*.

We suggest using models trained on the standard language modeling objective; as argued above, while models trained on RLHF or RLVR are interesting objects of study, models trained purely on distributional statistics are more appropriate operationalizations of the distributional hypothesis per se.

#### Prompting Regime.

Adapting human subjects experiments for language models is not always straightforward. There are a variety of ways to present information to language models which may have different strengths and weaknesses (Günther & Cassani, 2025) for their use to extract distributional baselines.

First, the form of the task itself might need to be transformed. Human experiments that involve explicit prediction (like the cloze task) or a continuous measure of online processing difficulty (like reading time or N400 amplitude), are the most straightforward to adapt. The model’s surprisal in response to the stimulus (or some critical part of it) can be compared to the human participants’ responses (Jones & Bergen, 2024b; Michaelov et al., 2022).

Other kinds of offline tasks, however, such as comprehension questions, grammaticality judgements, or similarity ratings, could be adapted in multiple ways. One option is to use a measure of likelihood of the stimulus itself to predict participants’ offline judgements. For instance, researchers could present a grammatical sentence and an ungrammatical sentence to the model separately, and use the surprisal of each sentence to predict the rate at which participants would judge one sentence to be more grammatical than the other (Hu et al., 2026). Alternatively, researchers can present both versions of the sentence together in a single prompt, along with a question asking which sentence is more grammatical. They can then treat the probability assigned to certain tokens (such as “A” or “B”) in the models’ response as indicative of the model’s bias toward that sentence being more grammatical (e.g., Dentella et al. (2023)). This approach is advantageous in that it is very flexible, arguably more similar to the human experiment, and presents all question options to the model simultaneously. On the other hand, it is more distant from the next-word prediction objective for which LLMs were originally trained, introduces response biases (Holtzman et al., 2021), and requires metalinguistic representations which might go beyond the sensitivity being tested (Hu & Levy, 2023; Hu et al., 2026).

Beyond the critical stimulus itself, researchers face choices around the kind of context to provide to the model. Few-shot prompting, where models are provided with several examples of accurate performance before the critical question, has been found to improve performance in many domains (Brown et al., 2020). While this might appear to introduce an asymmetry that could artificially inflate estimates of distributional predictability, human participants often complete practice trials or are given examples which provide the same information. Similarly, human participants are often presented with detailed instructions about how to respond to questions (e.g., definitions of key terms like “similarity” or “implies”). Researchers must decide whether to include the same instructions in the model’s context window (Trott, 2025; Trott et al., 2025). Finally, human participants often improve across experiments (even without feedback), due to more general learning, for example, about the distribution of differences between stimuli, which might provide information about what is being tested. Researchers could address this either by testing for order effects in human participants (Jones et al., 2024b), or by testing whether LLM performance improves when previous questions and the model’s own responses are iteratively appended to the context window (Rathi et al., 2024).

##### *Recommendation*.

For online processing tasks, we recommend using surprisal directly rather than prompt-based approaches, as the former avoids potential metalinguistic confounds (Hu & Frank, 2024; Song et al., 2025). We recommend incorporating a *prefix* (e.g., with instructions or examples) before the target stimulus where it plays a crucial role in the design of the experiment (e.g., as with an instructional manipulation), keeping in mind that closely matching the human experiment might provide more commensurable estimates, while a more minimalist approach might provide a stricter baseline for distributional explanations. Moreover, in the case of a prefix specifically, researchers should consider whether the model in question (particularly if the model has not been subject to instruction-tuning) can integrate the relevant instructions into its behavior or whether this introduces other metalinguistic confounds (Hu & Frank, 2024). In all cases, researchers should explicitly justify their chosen operationalization over alternatives, and ideally provide empirical estimates of the robustness of their results to these decisions (see Trott et al., 2026 for an example).

#### Degree of Openness.

Language models also vary in the degree to which information about their training data and parameters is publicly available or “open”. Unfortunately, many state-of-the-art LLMs are “closed”—little is known about their architectural details or training regime. This makes it difficult to select among closed-source models according to the desiderata we’ve described thus far. It also makes it difficult to rule out the possibility of data contamination, i.e., in which some proportion of the test set also appeared in a model’s training data (see also “Data Contamination” below). In the context of distributional baselines specifically, this could have the effect of improving model performance on certain psycholinguistic tasks, potentially leading to *false negatives* with respect to the alternative hypothesis in question^3^.

Additionally, models built and maintained by for-profit companies might be subject to deprecation or alteration that raise serious challenges for scientific reproducibility (Johnson & Obradovich, 2024). A related concern is that increasingly, closed models are not built with the goal of simulating psychologically plausible models of language; the companies building them are typically interested in building the most “useful” model for various tasks (e.g., computer programming), which often involves considerable fine-tuning or other post-training procedures (see also “Training Regime”). Of course, because the most performant models tend to be closed-source, relying solely on open-source models might lead to an underestimation of the information that is in principle extractable from distributional statistics, raising the probability of *false positives* for the alternative hypothesis.

##### *Recommendation*.

We recommend that researchers prioritize the use of open-source models as distributional baselines. If researchers do use closed-source models—e.g., to establish an upper-bound of distributional predictability—we suggest also testing the best available open-weight models, e.g., those available through HuggingFace; as argued above, both the validity and reproducibility of results obtained with closed-source models are questionable. See other work (Hussain et al., 2024; Liesenfeld & Dingemanse, 2024; Trott et al., 2026) for more extensive discussion of the relevant considerations and trade-offs involved.

#### Addressing Data Contamination.

A related concern is *data contamination*, i.e., when a model is tested on data it has previously been trained on. Because LLMs contain so many parameters and are trained on such massive datasets, they are able to memorize and recall verbatim specific strings of text from their training data (Carlini et al., 2021). Thus, contamination can inflate estimates of model performance, i.e., giving the impression of generalization when in fact the model is simply repeating the sequences it was trained on. Contamination might also result in simply inflating the probability of strings the model has observed in its training set, giving a biased estimate of distributional predictability and potentially affecting the calibration of human dependent measures to the distributional baseline. In principle, it is possible that some LLMs have been trained on publicly available experimental research stimuli. Below, we outline several approaches to dealing with this possibility.

First, researchers can craft entirely new stimuli that operationalize the construct in question (e.g., Trott et al., 2023). Sufficiently unlikely novel stimuli would have a low enough chance to appear in the training data to make new items a reasonable approach to avoiding *verbatim* data contamination, i.e., in which a model has been trained on sequences of string that fully overlap with a test set. (The question of how *different* an item needs to be from items in a training set to constitute true “generalization” is a matter of great philosophical and empirical interest, but we do not address it here.) Of course, this is also the most time-consuming and potentially costly method: in addition to creating new stimuli, researchers may need to *norm* them for various confounds.

Alternatively, researchers could avoid using stimuli that are likely to exist in the training data, especially as examples of the phenomenon of the question. For example, the string “colorless green ideas sleep furiously” is very likely to have appeared as an example of a grammatical but nonsensical sentence in a training corpus. If an LLM’s training data are open, researchers can conduct an *audit* of those data to search for examples of the stimuli they wish to use (see, e.g., Lampinen, 2024). Moreover, there is a growing number of methods for attempting to detect whether an LLM has observed a given string in its training data (e.g., Golchin & Surdeanu, 2023). These methods are by no means perfect, but they could act as an additional signal about whether to include certain items or not.

##### *Recommendation*.

The possibility of data contamination is a challenge for assessing whether LLMs are sensitive to a given experimental manipulation for the “right reasons”. In our view, the best way to avoid outright data contamination is to create entirely new stimuli; if researchers wish to use existing stimuli (e.g., for saving time, or for comparing against existing human datasets), we recommend that they conduct audits of the training data (if it is open) or use methods for detecting data contamination in LLMs directly (e.g., Golchin & Surdeanu, 2023); see also “Degree of Openness”.

#### “Grounded” Models.

A growing number of transformer language models are trained not only on text but also on other modalities, such as vision (Kim et al., 2021; Radford et al., 2021). Because these vision-language models (VLMs) are explicitly trained on non-linguistic input, they are not appropriate for testing the limits of what can be learned from language alone. However, they may still play a useful role in different kinds of psychological, psycholinguistic, and cognitive science research, e.g., as operationalizations of specific hypotheses about how distinct modalities are integrated; their psychometric predictive power can even be compared to that of text-only language models as a proxy for determining the role of sensorimotor grounding in human behavior (Bavaresco et al., 2024; Jones & Trott, 2024; Jones et al., 2024c).

##### *Recommendation*.

For testing hypotheses about the sufficiency of distributional statistics, we do not recommend using “grounded” models. If researchers are interested in hypotheses about multimodal input, grounded models are appropriate, though selecting among them involves additional considerations beyond our scope here.

#### Choice of Metric.

We have focused so far primarily on a specific operationalization of distributional predictability, i.e., *surprisal*, defined as the negative log probability of a word in context. The use of surprisal—as opposed to raw probability or alternative transformations of probability—is typically justified in two ways. First, surprisal enjoys some theoretical support as a relevant psycholinguistic variable, e.g., in surprisal theory (Aurnhammer & Frank, 2019; Hale, 2016; Levy, 2008; Smith & Levy, 2013). Second, language model surprisal (i.e., negative log-probability) has generally been found to be a better predictor of human dependent measures than raw probability (Michaelov & Bergen, 2024; Shain et al., 2024; Wilcox et al., 2020); though the same is not necessarily true of cloze probability (Brothers & Kuperberg, 2021; Michaelov & Bergen, 2024; Szewczyk & Federmeier, 2022), and it is also worth noting that a super-linear (Hoover et al., 2023; Meister et al., 2021) or sub-linear (Michaelov & Bergen, 2024) relationship between surprisal and the dependent measure can sometimes fit the data better rather than a strictly linear one (Smith & Levy, 2013).

Beyond measures of the predictability of particular words or word sequences in context, researchers have also explored other metrics derived from language models, such as those that involve directly probing the hidden states (or “embeddings”) from particular layers of a model. For example, as described in the Case Studies, Trott and Bergen (2023) calculated the cosine distance between contextualized representations of ambiguous English words in different contexts (e.g., “marinated lamb” vs. “friendly lamb”), then used this distance metric to predict human response time on a sensibility judgment task. The use of embeddings does raise additional methodological questions, including which layer of a model to select embeddings from, how embeddings should be aggregated across words and tokens, and which distance metric is used to calculate the similarity in a high-dimensional vector-space. These methodological concerns are beyond the scope of this paper.

##### *Recommendation*.

We recommend using surprisal (negative log-probability) as the default metric for operationalizing distributional predictability, for both the theoretical and empirical reasons described above. For tasks involving explicit judgments or comparisons between items (e.g., similarity judgments), the cosine distance between contextualized embeddings may be more appropriate; however, we recommend that researchers adopting this approach justify their choice of layer, aggregation method, and distance metric, and as with our analysis decisions (e.g., “Model Scale”), report sensitivity to these decisions.

## LIMITATIONS, OBJECTIONS, AND THE PATH FORWARD

The use of large language models as distributional baselines in the manner we propose is relatively new. As such, there are still many theoretical issues to address. In this section, we discuss potential objections to this approach, clarifying our own perspective on the role that LLMs can (and can’t) play in language processing research; we also outline a positive vision for the use of LLMs not only as *baselines* but as “model organisms” more generally.

### Objection: Necessity vs. Sufficiency

Suppose a metric derived from an LLM (e.g., surprisal) predicts human behavior in an experiment so well that an alternative theoretical variable of interest explains no additional variance. Does this entail that distributional predictability is the most plausible mechanism by which human comprehenders learn to represent or process the kinds of concepts tested in the experiment?

Our view is *no*. A distributional baseline can only establish the *sufficiency*—not the *necessity*—of distributional statistics in producing humanlike sensitivity to an experimental manipulation. That is, LLMs can be used as a “proof-of-concept” that, in principle, the behavior in question could have emerged from a distributional learner trained on a text corpus. Yet behavioral convergence between LLMs and humans need not emerge from the same mechanisms: indeed, distributional statistics from a representative corpus of human language reflect the product of human processing and conceptual knowledge (in addition to shaping it). But this sufficiency argument does entail a corresponding *non-necessity* of the theoretical concept in question. Put more concretely: the approach we advocate can only demonstrate that distributional statistics, say, are sufficient, but not necessary. However, by the same token, successful distributional predictability can disconfirm the necessity of the theoretical explanation. Now, in the case where distributional predictability and theoretical constructs each are sufficient to account for some data (but where neither is known to be necessary), the researchers find themselves in the familiar position of adjudicating on other criteria. For instance, researchers might ask about the respective theoretical plausibility of the models, i.e., their consistency with broader theory (as argued by Piantadosi et al., 2013), their pre-theoretical plausibility, their “tractability” as psycholinguistic theories (i.e., the ease of generating predictions from the account), or their predictive power (i.e., the range of situations in which the theory makes testable predictions) (Goldstein & Lederman, 2025). Or they might identify areas where the two models *do* make distinct predictions, using LLMs as part of the experimental design process in the manner described above, i.e., crafting “adversarial” stimuli to which LLMs and humans might show divergent behavior.

### Objection: How Generalizable is this Finding?

As noted above, available LLMs vary widely in their architecture, training data, and overall performance. Yet just as studies of human populations rely on *samples*, any study using LLMs as distributional baselines must select from the existing (and possible) “population” of language models. This raises two questions. First, to what extent can a finding obtained with a given *model instance* be generalized to a broader reference class of LLMs? And second, what is the implication of this for conclusions about the sufficiency of distributional statistics?

Unfortunately, the answer to the question of *when* a claim generalizes is still unknown: the study of LLMs is in its infancy and lacks coherent principles with which to guide generalizations even across broader “populations” of models (Trott, 2025), let alone to humans. Notably, this is also a challenge for the study of human populations, which has historically relied on unrepresentative samples (Henrich et al., 2010) in the search for general or “universal” mechanisms underlying human cognition (Kroupin et al., 2025). That said, there is a growing body of research comparing the psychometric predictive power of metrics from different language models (Michaelov & Bergen, 2022; Shain et al., 2024), and we anticipate the issue of generalizability across models becoming a primary research question if or when LLMs are adopted more broadly as distributional baselines.

The second-order question of *whether generalizability matters* depends upon the claim being made. If a researcher is using inductive reasoning to make a claim about the broader population of LLMs—i.e., when generalization is critical—then a sample of LLMs should be representative of the population that exists. But if the research is using *deductive reasoning* to disconfirm a null hypothesis—i.e., that distributional statistics does *not* produce sensitivity to some manipulation—then a single counterexample is sufficient. As argued above, we are advocating for the use of LLMs as sufficiency proofs: namely, that there exists (or does not exist) *at least one* model instance that exhibits sensitivity to the manipulation. In principle, of course, the null hypothesis under consideration could (and should) be iteratively refined depending on the goals of the research community, e.g., “No model trained with ≤X parameters trained on ≤Y tokens will exhibit sensitivity to this manipulation” (see also “Model Scale”). We note that this point also applies to concerns about the robustness of a given model’s behavior to different prompting techniques.

### Objection: Are LLMs *too* Good?

Another potential objection to the use of contemporary LLMs as baselines is that they are unrealistically good. A more precise form of this objection might be that LLMs are trained on too much data to be plausible models of distributional learning and are thus not informative baselines.

On the one hand, we agree that many LLMs are trained on developmentally implausible amounts of tokens, and we are excited about research on more plausible distributional learners, e.g., the BabyLM challenge (Warstadt et al., 2025). On the other hand, we disagree that this makes LLMs trained on an implausible number of tokens simply uninformative as baselines. If an LLM trained on language statistics alone (i.e., not subject to post-training such as RLHF) explains away an experimental variable, it serves to demonstrate that the relevant information is in principle extractable from the corpus; this opens the door to more targeted, precise tests that might involve more refined null hypotheses, e.g., those concerning the learnability of a representation or behavior from a specific volume, type, and order of input (see above). Conversely, if such a model does *not* explain away the experimental variable, it suggests that even an upper bound estimate of distributional learnability falls short, which should increase our confidence that distributional statistics are insufficient. More generally, no single study can fully “close the door” on a research question: as we argued in the “Best Practices” section, researchers might proceed by iteratively refining their theory and the baseline models under consideration, keeping in mind the relative costs of false positives and false negatives.

### Objection: Is Explaining Away All of The Variance Too High of a Bar?

In several of the case studies discussed above, distributional predictability was found to *partly* explain human behavior at a task (Jones & Bergen, 2024b; Jones et al., 2022; Trott, 2024). In these cases, the distributional measure was sensitive to the experimental manipulation and correlated with human judgements. However, the experimental manipulation explained additional variance in human behavior that could not be explained by the distributional predictor. In these cases, we claim the evidence suggests that distributional information is not sufficient to explain human behavior. But is explaining away *all* of the relevant variance (i.e., such that the experimental condition does not significantly improve model fit) too high of a bar? Isn’t sensitivity to the same manipulation enough to show that distributional predictability is sufficient in principle to explain the key mechanisms behind human behavior?

In favor of this objection, one could argue that the probability that LLMs assign to sequences are necessarily sensitive to various factors which might go beyond the construct being assessed (such as whether one group of sentences is more plausible than another). LLMs will be sensitive to the frequency of the individual words in the sentence, the typicality of the phrasing, as well as the way the sequence has been tokenized. We should not expect these factors to correlate perfectly with human behavior. More generally, LLMs are obviously not perfect models of distributional learning in human participants, having been exposed to totally different kinds of corpora. On this view, it would be taking the baseline account too seriously to expect an LLM to fully explain experimental effects on human comprehenders.

Overall, however, we *disagree* that explaining away all relevant variance is too high of a bar. First, LLM behavior does not have to perfectly correlate with human behavior in the baselines approach: it need only account for the extent to which human behavior is affected by the experimental manipulation. This means that idiosyncrasies (of either human or LLM processing) will impact the results only if they systematically vary with condition. Second, demonstrating sensitivity to a contrast is not enough to show that a confound could be responsible for a processing effect. Photovoltaic cells are sensitive to changes in brightness but this does not make them good models of the eye. Similarly, word frequency is correlated with many effects in psycholinguistics: we do not conclude that word frequency is driving these effects unless the manipulation explains no significant variance over and above word frequency.

Lastly, empirical evidence suggests that completely explaining away effects is possible in practice. Michaelov et al. (2022) found that LLM surprisal completely accounted for the effect of cloze probability on N400 amplitude. That the case studies show such predictive power for some phenomena (Michaelov et al., 2022), but not others such as word-sense boundaries (Trott & Bergen, 2023), pronoun resolution (Jones & Bergen, 2024b), or mental state reasoning (Jones et al., 2024; Trott et al., 2023), strengthens the case that information beyond distributional predictability is necessary in the latter processes.

### Objection: Surprisal is a Metric, Not a Mechanism

In the “Best Practices” section, we suggested the use of *surprisal*—the negative log probability assigned to a given token or token sequence—as a useful metric for operationalizing distributional predictability. As others have argued (Slaats & Martin, 2025), surprisal itself is a metric, not necessarily an explanatory variable that sheds light on the underlying mechanisms involved in language processing.

In some sense, we agree with this concern: any metric should be viewed in the context of the theoretical construct(s) it is intended to operationalize. While there are prominent theories that emphasize the information-theoretical properties of surprisal as helpful explanatory constructs for a computational-level understanding of the mind (e.g., Levy, 2008; Wilcox et al., 2023), our argument here does not depend on Surprisal Theory specifically. Our view is *not* that surprisal (or any other metric extracted from an LLM) is inherently informative about which representations or mechanisms underlie human language comprehension—or, for that matter, LLMs themselves. Similarly, measures of human behavior (e.g., reading time) or brain activity (e.g., EEG amplitude) are not inherently meaningful. Indeed, in virtually all areas of scientific investigation, a measured variable is interpretable only in the light of appropriate theoretical scaffolding and study design, even if that theoretical scaffolding is relatively minimal, e.g., an assumption that processing difficulty is correlated with reading time. Concretely: first, there must be a plausible link between some theoretical construct and the measured variable in question; and second, this variable must be analyzed in terms of some *other* theoretically meaningful variable, e.g., an experimental manipulation intended to shed light on a research question.

Surprisal, then, is *not* inherently meaningful in the absence of the appropriate theoretical scaffolding and experimental design. But it is a useful *index* into whether distributional predictability co-varies with some other variable, such as an experimental condition. More generally, this provides insight into whether sensitivity to an experimental manipulation can emerge purely from exposure to distributional statistics, which addresses key questions about the learnability of certain representations or behaviors from linguistic input alone. Finally, as discussed throughout, surprisal covaries with a number of human behavioral and neural responses, and also captures an operationalization of how probable a linguistic string is—these properties make it a useful metric for a distributional predictability baseline.

### Objection: Neural Language Models are Difficult to Interpret

Although language models are built and trained by humans, the *internal mechanisms* that give rise to their behavior are not easily interpretable from their architecture or parameters alone. This is why such models are occasionally called “opaque” or “black boxes”. Indeed, the field of “mechanistic interpretability” (Olah et al., 2020; Sharkey et al., 2025) exists primarily because of this opacity, and is dedicated to identifying the *circuits* and *representations* underlying behavior. This field has made some progress, e.g., in cataloging the putative functions of different *attention heads* (Edelman et al., 2024; Htut et al., 2019; Olsson et al., 2022), but a complete accounting (in either psychological or mechanistic terms) of LLM behavior remains elusive. Thus, one concern might be that even if distributional predictability succeeds in partially or fully explaining away an experimental manipulation, we will not be able to determine *why*. This is further complicated by the observation that distributional predictability likely reflects other relevant psycholinguistic variables, such as frequency, concreteness, or even age of acquisition, meaning that a “successful” baseline result cannot necessarily be attributed to distributional predictability per se. (See also “Surprisal is a metric, not a mechanism”, as well as “Are LLMs too good?”.)

Here, there are several important clarifications to make. First, although LLMs are opaque in many ways, we do—particularly with open-source models—have a good understanding of what they are trained to do (i.e., make predictions about upcoming tokens) and how they are trained to do it (e.g., which text corpora they are trained on, along with other decisions about training). Crucially, this “computational” level of understanding is sufficient for the argument we are advancing, which concerns the relation between certain kinds of input (e.g., language) and observable behavior (e.g., sensitivity to a particular experimental manipulation). It is common in Cognitive Science to make claims about the kinds of constructs that may causally influence each other without articulating the algorithmic or implementational details of how this influence is achieved; this is the level of analysis at which the “distributional baselines” method operates.

Relatedly, as we note elsewhere (see “Surprisal is a metric, not a mechanism”), our argument is not that a “successful” baselines result should be taken as confirmation that human behavior is, in fact, driven by distributional predictability. Rather, it is a proof-of-concept that distributional predictability is in principle *sufficient* to account for the effect of an experimental manipulation; this, in turn, serves as *disconfirmation* that the experimental effect in humans is necessarily driven by the intended construct the effect is taken to index (e.g., world knowledge). In this sense, the problem of a distributional confound is thus analogous to the problem of, say, a frequency confound: if the words in a lexical decision task vary in frequency across levels of an experimental manipulation, this confounds the theoretical inferences otherwise licensed by finding an experimental effect—given that there are both theoretical and empirical reasons to believe that unigram frequency is a relevant psycholinguistic variable.

Finally, the fact that distributional predictability may covary with other variables of interest (such as unigram frequency) is also not a fundamental problem for the baselines approach. As noted above, the goal of the baselines approach is to answer the following question: does an experimental manipulation account for variance in human behavior *above and beyond* that explained by distributional predictability? Answering this question is not undermined by the fact that surprisal correlates with other variables, *provided* the researcher is careful not to make specific mechanistic claims about surprisal as the particular causal mechanism driving human behavior. Our case is more modest: a “successful” baselines result does not provide evidence that surprisal *causes* an effect, but rather serves as an index of what is achievable through exposure to distributional statistics. Just as unigram frequency might constitute an alternative causal explanation for an effect, distributional predictability represents another such alternative. Moreover, the fact that surprisal correlates with other variables could be taken as a sign of its utility and predictive power. Of course, we do encourage researchers who wish to disentangle the effect of surprisal from other variables (such as frequency) to do so, e.g., as in Goodkind and Bicknell (2021).

### The Path Forward

We have argued that the *distributional predictability* of linguistic stimuli ought to be considered in the design and analysis of language processing experiments. As with other linguistic properties such as word frequency or concreteness, distributional predictability reliably co-varies with key dependent variables (e.g., reading time)—and in many cases offers an alternative, plausible explanatory route for the theoretical construct of interest. Moreover, it is now tractable to derive estimates of distributional predictability from statistical language models. Thus, statistical language models can be used as “baselines” for approximating which behaviors could in principle emerge from exposure to linguistic input alone.

Moving forward, we hope our proposal will contribute to further theoretical and methodological *refinement* of this approach. In this section and the “Best Practices” section, we have already enumerated some of what this might entail. Many theoretical and empirical issues must be addressed, including: the choice of metric and architecture; determining what amount of training data counts as “developmentally plausible”; and identifying which findings generalize from specific model instances to a broader “reference class”.

We also anticipate several more general applications of language models as distributional baselines. First, language models offer a degree of *control* and *access* that is impossible with human participants. In principle, researchers can control which data a model is trained on, allowing researchers to test questions about what kinds of input is required to form which kinds of generalizations (Misra & Mahowald, 2024; Yao et al., 2025); in some cases, this approach could even be used to generate *novel* hypotheses, which could in turn be tested in human participants (Misra & Kim, 2024). Similarly, the internal states of language models can be directly probed and even manipulated, allowing researchers to test questions about the representations and mechanisms that give rise to model behaviors (Marks et al., 2024; Olsson et al., 2022; Wang et al., 2022). While work on language model interpretability is still arguably “pre-paradigmatic” (Olah et al., 2020), it represents another promising direction for testing hypotheses that cannot be directly assessed in humans. In this way, language models could be used as a kind of “model organism” to establish convergent evidence for certain research questions in psycholinguistics.

This notion of language models as “model organisms” connects to another possible future direction, which expands beyond the use of *text-only* models to models trained on a variety of modalities. Naturally, a model trained on both text data and images (i.e., a “vision-language model” or VLM) should not be used as an operationalization of theories about linguistic input specifically. Yet these models could be used as implementations of distinct theories about whether and to what extent linguistic representations are grounded with non-linguistic information (Barsalou, 1999; Bergen, 2015; Glenberg & Kaschak, 2002). Indeed, recent work has already begun comparing the psychometric predictive power of text-only language models to models exposed to visual information, though in practice visual grounding does not always improve psychometric predictive power (Bavaresco et al., 2024; Bavaresco & Fernández, 2025; Jones et al., 2024c; Jones & Trott, 2024). In theory, modifying the training regime and architecture of these VLMs could allow researchers to test questions about multimodal conceptual integration in humans.

Human cognition clearly does not depend *solely* on the distributional statistics of language. But quantifying and controlling for the extent to which human behavior could be the product of distributional learning from language allows us to more precisely understand and attribute responsibility for the various behavioral and neural measures of cognitive processing. The advent of Large Language Models provides us with a tool to do exactly this, and while consulting distributional baselines is in its infancy, the practice stands to develop alongside the tools it makes use of.

## ACKNOWLEDGMENTS

Thank you to Victor Ferreira, Seana Coulson, Yoonwon Jung, Samuel Taylor, and Aaron Cohen for valuable discussion about the topics in this paper.

## AUTHOR CONTRIBUTIONS

Sean Trott: Conceptualization; Data curation; Formal analysis; Project administration; Visualization; Writing – original draft; Writing – review & editing. James Michaelov: Conceptualization; Writing – original draft; Writing – review & editing. Cameron Jones: Conceptualization; Visualization; Writing – original draft; Writing – review & editing. Tyler Chang: Conceptualization; Writing – original draft; Writing – review & editing. Benjamin Bergen: Conceptualization; Supervision; Writing – original draft; Writing – review & editing.

## DATA AND CODE AVAILABILITY STATEMENT

Demonstration code (and data) for the baselines method is included on GitHub: https://github.com/seantrott/baselines_demo/.

## Notes

^1^ In principle, the “distribution” of a word could also include its distribution among non-linguistic contexts (e.g., co-speech gestures, prosodic contours, social factors, etc.), but our focus here as with almost all distributional work is on operationalizing the *textual distribution* of a word.^2^ Note that tokens are generally assigned using compression algorithms such as byte-pair encoding (Sennrich et al., 2016) that allows the vocabulary of the model to effectively cover all possible text sequences, with more common sequences of bytes generally being merged into tokens. Thus, some (generally more common) words are represented by one token, while others are represented by sequences of sub-word tokens.^3^ I.e., *incorrectly* finding that a given LLM explains away the human effect.

## References

[bib1] Andrews, M., Vigliocco, G., & Vinson, D. (2009). Integrating experiential and distributional data to learn semantic representations. Psychological Review, 116(), 463–498. 10.1037/a0016261, 19618982

[bib2] Aurnhammer, C., & Frank, S. L. (2019). Evaluating information-theoretic measures of word prediction in naturalistic sentence reading. Neuropsychologia, 134, 107198. 10.1016/j.neuropsychologia.2019.107198, 31553896

[bib3] Barr, D. J., Levy, R., Scheepers, C., & Tily, H. J. (2013). Random effects structure for confirmatory hypothesis testing: Keep it maximal. Journal of Memory and Language, 68(3), 255–278. 10.1016/j.jml.2012.11.001, 24403724 PMC3881361

[bib4] Barsalou, L. W. (1999). Perceptual symbol systems. Behavioral and Brain Sciences, 22(4), 577–660. 10.1017/S0140525X99002149, 11301525

[bib5] Bates, D., Mächler, M., Bolker, B., & Walker, S. (2015). Fitting linear mixed-effects models using lme4. Journal of Statistical Software, 67, 1–48. 10.18637/jss.v067.i01

[bib6] Bavaresco, A., & Fernández, R. (2025). Experiential semantic information and brain alignment: Are multimodal models better than language models? arXiv. 10.48550/arXiv.2504.00942

[bib180] Bavaresco, A., de Heer Kloots, M., Pezzelle, S., & Fern´ndez, R. (2024). Vision-language models align with human neural representations in concept processing. arXiv. 10.48550/arXiv.2407.17914

[bib7] Bedny, M., Pascual-Leone, A., & Saxe, R. R. (2009). Growing up blind does not change the neural bases of Theory of Mind. Proceedings of the National Academy of Sciences, 106(27), 11312–11317. 10.1073/pnas.0900010106, 19553210 PMC2708685

[bib8] Bender, E. M., Gebru, T., McMillan-Major, A., & Shmitchell, S. (2021). On the dangers of stochastic parrots: Can language models be too big? In Proceedings of the 2021 ACM conference on fairness, accountability, and transparency (pp. 610–623). Association for Computing Machinery. 10.1145/3442188.3445922

[bib9] Bergen, B. (2015). Embodiment, simulation and meaning. In The Routledge handbook of semantics (pp. 142–157). Routledge.

[bib10] Boleda, G. (2020). Distributional semantics and linguistic theory. Annual Review of Linguistics, 6(1), 213–234. 10.1146/annurev-linguistics-011619-030303

[bib11] Brothers, T., & Kuperberg, G. R. (2021). Word predictability effects are linear, not logarithmic: Implications for probabilistic models of sentence comprehension. Journal of Memory and Language, 116, 104174. 10.1016/j.jml.2020.104174, 33100508 PMC7584137

[bib12] Brown, J. R., Donelan-McCall, N., & Dunn, J. (1996). Why talk about mental states? The significance of children's conversations with friends, siblings, and mothers. Child Development, 67(3), 836–849. 10.2307/1131864, 8706529

[bib13] Brown, T., Mann, B., Ryder, N., Subbiah, M., Kaplan, J. D., Dhariwal, P., & Amodei, D. (2020). Language models are few-shot learners. Advances in Neural Information Processing Systems, 33, 1877–1901.

[bib14] Burnham, K. P., Anderson, D. R., & Huyvaert, K. P. (2011). AIC model selection and multimodel inference in behavioral ecology: Some background, observations, and comparisons. Behavioral Ecology and Sociobiology, 65(1), 23–35. 10.1007/s00265-010-1029-6

[bib15] Carlini, N., Tramer, F., Wallace, E., Jagielski, M., Herbert-Voss, A., Lee, K., & Raffel, C. (2021). Extracting training data from large language models. In 30th USENIX security symposium (USENIX Security 21) (pp. 2633–2650).

[bib16] Connell, L. (2019). What have labels ever done for us? The linguistic shortcut in conceptual processing. Language, Cognition and Neuroscience, 34(10), 1308–1318. 10.1080/23273798.2018.1471512

[bib18] DeLong, K. A., Trott, S., & Kutas, M. (2023). Offline dominance and zeugmatic similarity normings of variably ambiguous words assessed against a neural language model (BERT). Behavior Research Methods, 55(4), 1537–1557. 10.3758/s13428-022-01869-6, 35689168 PMC10040203

[bib19] Dentella, V., Günther, F., & Leivada, E. (2023). Systematic testing of three language models reveals low language accuracy, absence of response stability, and a yes-response bias. Proceedings of the National Academy of Sciences, 120(51), e2309583120. 10.1073/pnas.2309583120, 38091290 PMC10743380

[bib20] Dove, G. (2020). More than a scaffold: Language is a neuroenhancement. Cognitive Neuropsychology, 37(5–6), 288–311. 10.1080/02643294.2019.1637338, 31269862

[bib181] Edelman, E., Tsilivis, N., Edelman, B. L., Malach, E., & Goel, S. (2024). The evolution of statistical induction heads: In-context learning Markov chains. Advances in neural information processing systems, 37, 642373–624311.

[bib21] Elman, J. L. (1990). Finding structure in time. Cognitive Science, 14(2), 179–211. 10.1207/s15516709cog1402_1

[bib22] Elman, J. L. (2009). On the meaning of words and dinosaur bones: Lexical knowledge without a lexicon. Cognitive Science, 33(4), 547–582. 10.1111/j.1551-6709.2009.01023.x, 19662108 PMC2721468

[bib23] Ettinger, A. (2020). What BERT is not: Lessons from a new suite of psycholinguistic diagnostics for language models. Transactions of the Association for Computational Linguistics, 8, 34–48. 10.1162/tacl_a_00298

[bib24] Federmeier, K. D. (2022). Connecting and considering: Electrophysiology provides insights into comprehension. Psychophysiology, 59(1), e13940. 10.1111/psyp.13940, 34520568 PMC9009268

[bib25] Firth, J. R. (1957). A synopsis of linguistic theory, 1930–1955. In J. R. Firth (Ed.), Studies in linguistic analysis (pp. 1–32). Blackwell.

[bib26] Frank, S. L., & Bod, R. (2011). Insensitivity of the human sentence-processing system to hierarchical structure. Psychological Science, 22(6), 829–834. 10.1177/0956797611409589, 21586764

[bib27] Frank, S. L., Fernandez Monsalve, I., Thompson, R. L., & Vigliocco, G. (2013). Reading time data for evaluating broad-coverage models of English sentence processing. Behavior Research Methods, 45(4), 1182–1190. 10.3758/s13428-012-0313-y, 23404612

[bib28] Frank, S. L., Otten, L. J., Galli, G., & Vigliocco, G. (2015). The ERP response to the amount of information conveyed by words in sentences. Brain and Language, 140, 1–11. 10.1016/j.bandl.2014.10.006, 25461915

[bib29] Futrell, R., Wilcox, E., Morita, T., Qian, P., Ballesteros, M., & Levy, R. (2019). Neural language models as psycholinguistic subjects: Representations of syntactic state. arXiv. 10.48550/arXiv.1903.03260

[bib30] Gelman, A., & Hill, J. (2007). Data analysis using regression and multilevel/hierarchical models. Cambridge University Press.

[bib31] Glenberg, A. M., & Kaschak, M. P. (2002). Grounding language in action. Psychonomic Bulletin & Review, 9(3), 558–565. 10.3758/BF03196313, 12412897

[bib32] Glenberg, A. M., & Robertson, D. A. (2000). Symbol grounding and meaning: A comparison of high-dimensional and embodied theories of meaning. Journal of Memory and Language, 43(3), 379–401. 10.1006/jmla.2000.2714

[bib33] Golchin, S., & Surdeanu, M. (2023). Time travel in LLMs: Tracing data contamination in large language models. arXiv. 10.48550/arXiv.2308.08493

[bib34] Goldstein, S., & Lederman, H. (2025). What does ChatGPT want? An interpretationist guide.

[bib35] Goodkind, A., & Bicknell, K. (2021). Local word statistics affect reading times independently of surprisal. arXiv. 10.48550/arXiv.2103.04469

[bib36] Graesser, A. C., Singer, M., & Trabasso, T. (1994). Constructing inferences during narrative text comprehension. Psychological Review, 101(3), 371–395. 10.1037/0033-295X.101.3.371, 7938337

[bib37] Grieve, J., Bartl, S., Fuoli, M., Grafmiller, J., Huang, W., Jawerbaum, A., Murakami, A., Perlman, M., Roemling, D., & Winter, B. (2025). The sociolinguistic foundations of language modeling. Frontiers in Artificial Intelligence, 7, 1472411. 10.3389/frai.2024.1472411, 39871863 PMC11770026

[bib38] Günther, F., & Cassani, G. (2025). Large language models in psycholinguistic studies. 10.31234/osf.io/cvnam_v1

[bib39] Günther, F., Dudschig, C., & Kaup, B. (2018). Symbol grounding without direct experience: Do words inherit sensorimotor activation from purely linguistic context? Cognitive Science, 42, 336–374. 10.1111/cogs.12549, 29052241

[bib40] Haber, J., & Poesio, M. (2024). Polysemy—Evidence from linguistics, behavioral science, and contextualized language models. Computational Linguistics, 50(1), 351–417. 10.1162/coli_a_00500

[bib41] Hale, C. M., & Tager-Flusberg, H. (2003). The influence of language on theory of mind: A training study. Developmental Science, 6(3), 346–359. 10.1111/1467-7687.00289, 16467908 PMC1350918

[bib42] Hale, J. (2016). Information-theoretical complexity metrics. Language and Linguistics Compass, 10(9), 397–412. 10.1111/lnc3.12196

[bib43] Harris, Z. (1954). Distributional structure. Word, 10(23), 146–162. 10.1080/00437956.1954.11659520

[bib44] Hart, B., & Risley, T. R. (1992). American parenting of language-learning children: Persisting differences in family-child interactions observed in natural home environments. Developmental Psychology, 28(6), 1096. 10.1037/0012-1649.28.6.1096

[bib45] Henrich, J., Heine, S. J., & Norenzayan, A. (2010). The weirdest people in the world? Behavioral and Brain Sciences, 33(2–3), 61–83. 10.1017/S0140525X0999152X, 20550733

[bib46] Hobbs, J. R. (1979). Coherence and coreference. Cognitive Science, 3(1), 67–90. 10.1207/s15516709cog0301_4

[bib47] Holtzman, A., West, P., Shwartz, V., Choi, Y., & Zettlemoyer, L. (2021). Surface form competition: Why the highest probability answer isn't always right. arXiv. 10.48550/arXiv.2104.08315

[bib48] Hoover, J. L., Sonderegger, M., Piantadosi, S. T., & O’Donnell, T. J. (2023). The plausibility of sampling as an algorithmic theory of sentence processing. Open Mind: Discoveries in Cognitive Science, 7, 350–391. 10.1162/opmi_a_00086, 37637302 PMC10449406

[bib49] Hosseini, E. A., Schrimpf, M., Zhang, Y., Bowman, S., Zaslavsky, N., & Fedorenko, E. (2024). Artificial neural network language models predict human brain responses to language even after a developmentally realistic amount of training. Neurobiology of Language, 5(1), 43–63. 10.1162/nol_a_00137, 38645622 PMC11025646

[bib50] Htut, P. M., Phang, J., Bordia, S., & Bowman, S. R. (2019). Do attention heads in BERT track syntactic dependencies? arXiv. 10.48550/arXiv.1911.12246

[bib51] Hu, J., Floyd, S., Jouravlev, O., Fedorenko, E., & Gibson, E. (2023). A fine-grained comparison of pragmatic language understanding in humans and language models. In Proceedings of the 61st annual meeting of the association for computational linguistics (Volume 1: Long papers) (pp. 4194–4213). Association for Computational Linguistics. 10.18653/v1/2023.acl-long.230

[bib52] Hu, J., & Frank, M. C. (2024). Auxiliary task demands mask the capabilities of smaller language models. First Conference on Language Modeling.

[bib53] Hu, J., & Levy, R. (2023). Prompting is not a substitute for probability measurements in large language models. arXiv. 10.48550/arXiv.2305.13264

[bib54] Hu, J., Mahowald, K., Lupyan, G., Ivanova, A., & Levy, R. (2024). Language models align with human judgments on key grammatical constructions. Proceedings of the National Academy of Sciences, 121(36), e2400917121. 10.1073/pnas.2400917121, 39186652 PMC11388428

[bib55] Hu, J., Wilcox, E. G., Song, S., Mahowald, K., & Levy, R. P. (2026). What can string probability tell us about grammaticality? Transactions of the Association for Computational Linguistics, 14, 124–146. 10.1162/TACL.a.611

[bib56] Huang, K. J., Arehalli, S., Kugemoto, M., Muxica, C., Prasad, G., Dillon, B., & Linzen, T. (2024). Large-scale benchmark yields no evidence that language model surprisal explains syntactic disambiguation difficulty. Journal of Memory and Language, 137, 104510. 10.1016/j.jml.2024.104510

[bib57] Hughes, C., & Ensor, R. (2005). Executive function and theory of mind in 2 year olds: A family affair? Developmental Neuropsychology, 28(2), 645–668. 10.1207/s15326942dn2802_5, 16144431

[bib58] Hussain, Z., Binz, M., Mata, R., & Wulff, D. U. (2024). A tutorial on open-source large language models for behavioral science. Behavior Research Methods, 56(8), 8214–8237. 10.3758/s13428-024-02455-8, 39147947 PMC11525391

[bib59] Johnson, T., & Obradovich, N. (2024). Measuring an artificial intelligence language model’s trust in humans using machine incentives. Journal of Physics: Complexity, 5(1), 015003. 10.1088/2632-072X/ad1c69

[bib60] Jones, C., & Bergen, B. (2024a). Does GPT-4 pass the Turing test? In Proceedings of the 2024 conference of the North American chapter of the association for computational linguistics: Human language technologies (Volume 1: Long Papers) (pp. 5183–5210). Association for Computational Linguistics. 10.18653/v1/2024.naacl-long.290

[bib61] Jones, C. R., & Bergen, B. (2021). The role of physical inference in pronoun resolution. In Proceedings of the Annual Meeting of the Cognitive Science Society (Vol. 43, No. 43).

[bib62] Jones, C. R., & Bergen, B. (2024b). Does word knowledge account for the effect of world knowledge on pronoun interpretation? Language and Cognition, 16(4), 1182–1213. 10.1017/langcog.2024.2

[bib63] Jones, C. R., Bergen, B., & Trott, S. (2024c). Do multimodal large language models and humans ground language similarly? Computational Linguistics, 50(4), 1415–1440. 10.1162/coli_a_00531

[bib64] Jones, C. R., Chang, T. A., Coulson, S., Michaelov, J. A., Trott, S., & Bergen, B. (2022). Distrubutional semantics still can't account for affordances. In Proceedings of the annual meeting of the cognitive science society (Vol. 44, No. 44).

[bib65] Jones, C. R., & Trott, S. (2024). Multimodal language models show evidence of embodied simulation. In Proceedings of the 2024 joint international conference on computational linguistics, language resources and evaluation (LREC-COLING 2024) (pp. 11928–11933). ELRA and ICCL.

[bib66] Jones, C. R., Trott, S., & Bergen, B. (2024a). Comparing humans and large language models on an Experimental Protocol Inventory for Theory of Mind Evaluation (EPITOME). Transactions of the Association for Computational Linguistics, 12, 803–819. 10.1162/tacl_a_00674

[bib67] Jones, C. R., Trott, S., & Bergen, B. (2024b). Does reading words help you to read minds? A comparison of humans and LLMs at a recursive mindreading task. In Proceedings of the annual meeting of the cognitive science society (Vol. 46).

[bib68] Jurafsky, D., & Martin, J. H. (2025). Speech and language processing: An introduction to natural language processing, computational linguistics, and speech recognition with language models (3rd ed.) [Online manuscript]. Stanford University. https://web.stanford.edu/~jurafsky/slp3

[bib69] Kaplan, J., McCandlish, S., Henighan, T., Brown, T. B., Chess, B., Child, R., Gray, S., Radford, A., Wu, J., & Amodei, D. (2020). Scaling laws for neural language models. arXiv. 10.48550/arXiv.2001.08361

[bib70] Kauf, C., & Ivanova, A. A. (2023). A better way to do masked language model scoring. In Proceedings of the 61st annual meeting of the association for computational linguistics (Volume 2: Short Papers) (pp. 925–935). Association for Computational Linguistics. 10.18653/v1/2023.acl-short.80

[bib71] Kauf, C., Ivanova, A. A., Rambelli, G., Chersoni, E., She, J. S., Chowdhury, Z., Fedorenko, E., & Lenci, A. (2023). Event knowledge in large language models: The gap between the impossible and the unlikely. Cognitive Science, 47(11), e13386. 10.1111/cogs.13386, 38009752

[bib72] Kim, W., Son, B., & Kim, I. (2021). Vilt: Vision-and-language transformer without convolution or region supervision. In International conference on machine learning (pp. 5583–5594). PMLR.

[bib73] Kocijan, V., Davis, E., Lukasiewicz, T., Marcus, G., & Morgenstern, L. (2023). The defeat of the winograd schema challenge. Artificial Intelligence, 325, 103971. 10.1016/j.artint.2023.103971

[bib74] Krieger, B., Brouwer, H., Aurnhammer, C., & Crocker, M. W. (2025). On the limits of LLM surprisal as a functional explanation of the N400 and P600. Brain Research, 1865, 149841. 10.1016/j.brainres.2025.149841, 40782952

[bib75] Kroupin, I., Davis, H. E., & Henrich, J. (2025). Beyond newton: Why assumptions of universality are critical to cognitive science, and how to finally move past them. Psychological Review, 132(2), 291–310. 10.1037/rev0000480, 38753386

[bib76] Kuperberg, G. R., Brothers, T., & Wlotko, E. W. (2020). A tale of two positivities and the N400: Distinct neural signatures are evoked by confirmed and violated predictions at different levels of representation. Journal of Cognitive Neuroscience, 32(1), 12–35. 10.1162/jocn_a_01465, 31479347 PMC7299186

[bib77] Kuribayashi, T., Oseki, Y., Ito, T., Yoshida, R., Asahara, M., & Inui, K. (2021). Lower perplexity is not always human-like. In Proceedings of the 59th Annual Meeting of the Association for Computational Linguistics and the 11th International Joint Conference on natural Language Processing (Volume 1: Long Papers) (pp. 5203–5217). Association for Computational Linguistics. 10.18653/v1/2021.acl-long.405

[bib78] Kutas, M., & Federmeier, K. D. (2011). Thirty years and counting: Finding meaning in the N400 component of the event-related brain potential (ERP). Annual Review of Psychology, 62(1), 621–647. 10.1146/annurev.psych.093008.131123, 20809790 PMC4052444

[bib79] Kutas, M., & Hillyard, S. A. (1980). Reading senseless sentences: Brain potentials reflect semantic incongruity. Science, 207(4427), 203–205. 10.1126/science.7350657, 7350657

[bib80] Lampinen, A. (2024). Can language models handle recursively nested grammatical structures? A case study on comparing models and humans. Computational Linguistics, 50(4), 1441–1476. 10.1162/coli_a_00525

[bib81] Landauer, T. K., & Dumais, S. T. (1997). A solution to Plato’s problem: The latent semantic analysis theory of acquisition, induction, and representation of knowledge. Psychological Review, 104(2), 211–240. 10.1037/0033-295X.104.2.211

[bib82] Lee, H., Phatale, S., Mansoor, H., Lu, K. R., Mesnard, T., Ferret, J., & Rastogi, A. (2023). Rlaif: Scaling reinforcement learning from human feedback with AI feedback.

[bib83] Lenci, A. (2008). Distributional semantics in linguistic and cognitive research. Italian Journal of Linguistics, 20(1), 1–31.

[bib84] Levy, R. (2008). Expectation-based syntactic comprehension. Cognition, 106(3), 1126–1177. 10.1016/j.cognition.2007.05.006, 17662975

[bib85] Levy, R. (2013). Memory and surprisal in human sentence comprehension. In Sentence processing (pp. 78–114). Psychology Press.

[bib86] Levy, R., Fedorenko, E., & Gibson, E. (2013). The syntactic complexity of Russian relative clauses. Journal of Memory and Language, 69(4), 461–495. 10.1016/j.jml.2012.10.005, 24711687 PMC3975271

[bib87] Lewis, M., Zettersten, M., & Lupyan, G. (2019). Distributional semantics as a source of visual knowledge. Proceedings of the National Academy of Sciences, 116(39), 19237–19238. 10.1073/pnas.1910148116, 31488726 PMC6765286

[bib88] Li, J., & Joanisse, M. F. (2021). Word senses as clusters of meaning modulations: A computational model of polysemy. Cognitive Science, 45(4), e12955. 10.1111/cogs.12955, 33873247

[bib89] Liesenfeld, A., & Dingemanse, M. (2024). Rethinking open source generative AI: Open-washing and the EU AI Act. In Proceedings of the 2024 ACM conference on fairness, accountability, and transparency (pp. 1774–1787). Association for Computing Machinery. 10.1145/3630106.3659005

[bib90] Linzen, T., Dupoux, E., & Goldberg, Y. (2016). Assessing the ability of LSTMs to learn syntax-sensitive dependencies. Transactions of the Association for Computational Linguistics, 4, 521–535. 10.1162/tacl_a_00115

[bib91] Linzen, T., & Leonard, B. (2018). Distinct patterns of syntactic agreement errors in recurrent networks and humans. In Proceedings of the annual meeting of the cognitive science society (Vol. 40).

[bib92] Louwerse, M. M. (2018). Knowing the meaning of a word by the linguistic and perceptual company it keeps. Topics in Cognitive Science, 10(3), 573–589. 10.1111/tops.12349, 29851286

[bib93] Louwerse, M. M., & Jeuniaux, P. (2010). The linguistic and embodied nature of conceptual processing. Cognition, 114(1), 96–104. 10.1016/j.cognition.2009.09.002, 19818435

[bib94] Lupyan, G., Gentry, H., & Zettersten, M. (2026). How important is language for human-like intelligence? Perspectives on Psychological Science, 21(1), 115–120. 10.1177/17456916251398539, 41499467

[bib95] Lupyan, G., & Winter, B. (2018). Language is more abstract than you think, or, why aren't languages more iconic? Philosophical Transactions of the Royal Society B: Biological Sciences, 373(1752), 20170137. 10.1098/rstb.2017.0137, 29915005 PMC6015821

[bib96] Marks, L., Paren, A., Krueger, D., & Barez, F. (2024). Enhancing neural network interpretability with feature-aligned sparse autoencoders. arXiv. 10.48550/arXiv.2411.01220

[bib97] Martínez, G., Molero, J. D., González, S., Conde, J., Brysbaert, M., & Reviriego, P. (2024). Using large language models to estimate features of multi-word expressions: Concreteness, valence, arousal. Behavior Research Methods, 57(1), 5. 10.3758/s13428-024-02515-z, 39633225

[bib98] McDonald, S., & Ramscar, M. (2001). Testing the distributional hypothesis: The influence of context on judgements of semantic similarity. In Proceedings of the Annual Meeting of the Cognitive Science Society (Vol. 23, No. 23).

[bib99] McDonald, S. A., & Shillcock, R. C. (2003). Eye movements reveal the on-line computation of lexical probabilities during reading. Psychological Science, 14(6), 648–652. 10.1046/j.0956-7976.2003.psci_1480.x, 14629701

[bib100] Meister, C., Pimentel, T., Haller, P., Jäger, L., Cotterell, R., & Levy, R. (2021). Revisiting the uniform information density hypothesis. In Proceedings of the 2021 Conference on Empirical Methods in Natural Language Processing (pp. 963–980). Association for Computational Linguistics. 10.18653/v1/2021.emnlp-main.74

[bib101] Merkx, D., & Frank, S. L. (2021). Human sentence processing: Recurrence or attention? In Proceedings of the Workshop on Cognitive Modeling and Computational Linguistics (pp. 12–22). Association for Computational Linguistics. 10.18653/v1/2021.cmcl-1.2

[bib182] Michaelov, J., & Bergen, B. (2022). The more human-like the language model, the more surprisal is the best predictor of N400 amplitude. In NeurIPS 2022 workshop on information-theoretic principles in cognitive systems.

[bib103] Michaelov, J. A., Arnett, C., & Bergen, B. K. (2024). Revenge of the fallen? Recurrent models match transformers at predicting human language comprehension metrics. In First conference on language modeling.

[bib102] Michaelov, J. A., & Bergen, B. K. (2020). How well does surprisal explain N400 amplitude under different experimental conditions? In Proceedings of the 24th Conference on Computational natural Language Learning (pp. 652–663). Association for Computational Linguistics. 10.18653/v1/2020.conll-1.53

[bib104] Michaelov, J. A., Bardolph, M. D., Coulson, S., & Bergen, B. (2021). Different kinds of cognitive plausibility: Why are transformers better than RNNs at predicting N400 amplitude? In Proceedings of the annual meeting of the cognitive science society (Vol. 43, No. 43).

[bib105] Michaelov, J. A., Bardolph, M. D., Van Petten, C. K., Bergen, B. K., & Coulson, S. (2024). Strong prediction: Language model surprisal explains multiple N400 effects. Neurobiology of Language, 5(1), 107–135. 10.1162/nol_a_00105, 38645623 PMC11025652

[bib107] Michaelov, J. A., & Bergen, B. K. (2024). On the mathematical relationship between contextual probability and N400 amplitude. Open Mind: Discoveries in Cognitive Science, 8, 859–897. 10.1162/opmi_a_00150, 39077107 PMC11285424

[bib108] Michaelov, J. A., & Bergen, B. K. (2026). Better language models better model the N400, but not reading time. Journal of Memory and Language, 149, 104762. 10.1016/j.jml.2026.104762

[bib109] Michaelov, J. A., Coulson, S., & Bergen, B. K. (2022). So cloze yet so far: N400 amplitude is better predicted by distributional information than human predictability judgements. IEEE Transactions on Cognitive and Developmental Systems, 15(3), 1033–1042. 10.1109/TCDS.2022.3176783

[bib110] Michaelov, J. A., Estacio, R., Zhang, Z., & Bergen, B. K. (2025). Not quite Sherlock Holmes: Language model predictions do not reliably differentiate impossible from improbable events. In Findings of the association for computational linguistics: ACL 2025, (pp. 13528–13551). Association for Computational Linguistics. 10.18653/v1/2025.findings-acl.696

[bib111] Mikolov, T., Chen, K., Corrado, G., & Dean, J. (2013). Efficient estimation of word representations in vector space. arXiv. 10.48550/arXiv.1301.3781

[bib112] Misra, K. (2022). minicons: Enabling flexible behavioral and representational analyses of transformer language models. arXiv. 10.48550/arXiv.2203.13112

[bib113] Misra, K., Ettinger, A., & Rayz, J. (2020). Exploring BERT’s sensitivity to lexical cues using tests from semantic priming. In Findings of the association for computational linguistics: EMNLP 2020 (pp. 4625–4635). Association for Computational Linguistics. 10.18653/v1/2020.findings-emnlp.415

[bib114] Misra, K., & Kim, N. (2024). Generating novel experimental hypotheses from language models: A case study on cross-dative generalization. arXiv.

[bib115] Misra, K., & Mahowald, K. (2024). Language models learn rare phenomena from less rare phenomena: The Case of the Missing AANNs. In Proceedings of the 2024 conference on empirical methods in natural language processing (pp. 913–929). Association for Computational Linguistics. 10.18653/v1/2024.emnlp-main.53

[bib116] Nair, S., Srinivasan, M., & Meylan, S. (2020). Contextualized word embeddings encode aspects of human-like word sense knowledge. arXiv. 10.48550/arXiv.2010.13057

[bib117] Nicenboim, B., Schad, D. J., & Vasishth, S. (2025). Introduction to Bayesian data analysis for cognitive science. CRC Press. 10.1201/9780429342646

[bib183] Nieuwland, M. S., Arkhipova, Y., & Rodríguez-Gómez, P. (2020). Anticipating words during spoken discourse comprehension: A large-scale, pre-registered replication study using brain potentials. Cortex, 133, 1–36. 10.1016/j.cortex.2020.09.007, 33096395 PMC7526661

[bib118] Oh, B.-D., Clark, C., & Schuler, W. (2022). Comparison of structural parsers and neural language models as surprisal estimators. Frontiers in Artificial Intelligence, 5, 777963. 10.3389/frai.2022.777963, 35310956 PMC8929193

[bib119] Oh, B.-D., & Schuler, W. (2023). Why does surprisal from larger transformer-based language models provide a poorer fit to human reading times? Transactions of the Association for Computational Linguistics, 11, 336–350. 10.1162/tacl_a_00548

[bib120] Oh, B.-D., Yue, S., & Schuler, W. (2024). Frequency explains the inverse correlation of large language models’ size, training data amount, and surprisal's fit to reading times. arXiv. 10.48550/arXiv.2402.02255

[bib121] Olah, C., Cammarata, N., Schubert, L., Goh, G., Petrov, M., & Carter, S. (2020). Zoom in: An introduction to circuits. Distill, 5(3), e00024–e00001. 10.23915/distill.00024.001

[bib122] Olsson, C., Elhage, N., Nanda, N., Joseph, N., DasSarma, N., Henighan, T., Mann, B., Askell, A., Bai, Y., Chen, A., Conerly, T., Drain, D., Ganguli, D., Hatfield-Dodds, Z., Hernandez, D., Johnston, S., Jones, A., Kernion, J., Lovitt, L., … Olah, C. (2022). In-context learning and induction heads. arXiv. 10.48550/arXiv.2209.11895

[bib123] Ong, D. C. (2024). GPT-ology, computational models, silicon sampling: How should we think about LLMs in cognitive science? arXiv. 10.48550/arXiv.2406.09464

[bib124] Ouyang, L., Boroditsky, L., & Frank, M. C. (2017). Semantic coherence facilitates distributional learning. Cognitive Science, 41, 855–884. 10.1111/cogs.12360, 26988338

[bib125] Ouyang, L., Wu, J., Jiang, X., Almeida, D., Wainwright, C. L., Mishkin, P., Zhang, C., Agarwal, S., Slama, K., Ray, A., Schulman, J., Hilton, J., Kelton, F., Miller, L., Simens, M., Askell, A., Welinder, P., Christiano, P., Leike, J., & Lowe, R. (2022). Training language models to follow instructions with human feedback. Advances in Neural Information Processing Systems, 35, 27730–27744.

[bib126] Parviz, M., Johnson, M., Johnson, B., & Brock, J. (2011). Using language models and latent semantic analysis to characterise the N400m neural response. In Proceedings of the Australasian language technology association workshop 2011 (pp. 38–46).

[bib127] Piantadosi, S. T., Tily, H., & Gibson, E. (2013). Information content versus word length in natural language: A reply to ferrer-i-cancho and moscoso del prado martin. arXiv. 10.48550/arXiv.1307.672

[bib128] R Core Team. (2021). R: A language and environment for statistical computing. R Foundation for Statistical Computing. https://www.R-project.org/

[bib129] Rabs, E., Delogu, F., Drenhaus, H., & Crocker, M. W. (2022). Situational expectancy or association? The influence of event knowledge on the N400. Language, Cognition and Neuroscience, 37(6), 766–784. 10.1080/23273798.2021.2022171

[bib130] Radford, A., Kim, J. W., Hallacy, C., Ramesh, A., Goh, G., Agarwal, S., & Sutskever, I. (2021). Learning transferable visual models from natural language supervision. In International conference on machine learning (pp. 8748–8763).

[bib131] Radford, A., Wu, J., Child, R., Luan, D., Amodei, D., & Sutskever, I. (2019). Language models are unsupervised multitask learners. OpenAI Blog, 1(8), 9.

[bib132] Rathi, I., Taylor, S., Bergen, B. K., & Jones, C. R. (2024). GPT-4 is judged more human than humans in displaced and inverted Turing tests. arXiv. 10.48550/arXiv.2407.08853

[bib184] Rayner, K., & Bertera, J. H. (1979). Reading without a fovea. Science, 206(4417), 468–469. 10.1126/science.504987, 504987

[bib133] Rivière, P. D., Beatty-Martínez, A. L., & Trott, S. (2025). Evaluating contextualized representations of (Spanish) ambiguous words: A new lexical resource and empirical analysis. In Proceedings of the 2025 conference of the nations of the Americas chapter of the association for computational linguistics: Human language technologies (Volume 1: Long Papers) (pp. 8322–8338).

[bib134] Rivière, P. D., & Trott, S. (2025). Start making sense(s): A developmental probe of attention specialization using lexical ambiguity. arXiv. 10.48550/arXiv.2511.21974

[bib135] Rohde, H., Futrell, R., & Lucas, C. G. (2021). What's new? A comprehension bias in favor of informativity. Cognition, 209, 104491. 10.1016/j.cognition.2020.104491, 33545512

[bib136] Samuel, D. (2024). BERTs are generative in-context learners. Advances in Neural Information Processing Systems, 37, 2558–2589.

[bib137] Sanford, A. J., & Garrod, S. C. (1998). The role of scenario mapping in text comprehension. Discourse Processes, 26(2–3), 159–190. 10.1080/01638539809545043

[bib138] Schrimpf, M., Blank, I. A., Tuckute, G., Kauf, C., Hosseini, E. A., Kanwisher, N., Tenenbaum, B., & Fedorenko, E. (2021). The neural architecture of language: Integrative modeling converges on predictive processing. Proceedings of the National Academy of Sciences, 118(45), e2105646118. 10.1073/pnas.2105646118, 34737231 PMC8694052

[bib139] Sennrich, R., Haddow, B., & Birch, A. (2016). Neural machine translation of rare words with subword units. In Proceedings of the 54th annual meeting of the association for computational linguistics (Volume 1: Long papers) (pp. 1715–1725).

[bib140] Shain, C. (2019). A large-scale study of the effects of word frequency and predictability in naturalistic reading. In Proceedings of the 2019 conference of the North American chapter of the association for computational linguistics: Human language technologies, Volume 1 (Long and Short papers) (pp. 4086–4094). Association for Computational Linguistics. 10.18653/v1/N19-1413

[bib141] Shain, C., Meister, C., Pimentel, T., Cotterell, R., & Levy, R. (2024). Large-scale evidence for logarithmic effects of word predictability on reading time. Proceedings of the National Academy of Sciences, 121(10), e2307876121. 10.1073/pnas.2307876121, 38422017 PMC10927576

[bib142] Shao, Z., Wang, P., Zhu, Q., Xu, R., Song, J., Bi, X., Zhang, H., Zhang, M., Li, Y. K., Wu, Y., & Guo, D. (2024). Deepseekmath: Pushing the limits of mathematical reasoning in open language models. arXiv. 10.48550/arXiv.2402.03300

[bib143] Sharkey, L., Chughtai, B., Batson, J., Lindsey, J., Wu, J., Bushnaq, L., Goldowsky-Dill, N., Heimersheim, S., Ortega, A., Bloom, J., Biderman, S., Garriga-Alonso, A., Conmy, A., Nanda, N., Rumbelow, J., Wattenberg, M., Schoots, N., Miller, J., Michaud, E. J., … McGrath, T. (2025). Open problems in mechanistic interpretability. arXiv. 10.48550/arXiv.2501.16496

[bib144] Slaats, S., & Martin, A. E. (2025). What’s surprising about surprisal. Computational Brain & Behavior, 8(2), 233–248. 10.1007/s42113-025-00237-9, 40453151 PMC12125142

[bib145] Smith, N. J., & Levy, R. (2008). Optimal processing times in reading: A formal model and empirical investigation. In Proceedings of the annual meeting of the cognitive science society (Vol. 30, No. 30).

[bib146] Smith, N. J., & Levy, R. (2013). The effect of word predictability on reading time is logarithmic. Cognition, 128(3), 302–319. 10.1016/j.cognition.2013.02.013, 23747651 PMC3709001

[bib147] Song, S., Hu, J., & Mahowald, K. (2025). Language models fail to introspect about their knowledge of language. arXiv. 10.48550/arXiv.2503.07513

[bib148] Staub, A. (2011). The effect of lexical predictability on distributions of eye fixation durations. Psychonomic Bulletin & Review, 18(2), 371–376. 10.3758/s13423-010-0046-9, 21327339

[bib149] Steegen, S., Tuerlinckx, F., Gelman, A., & Vanpaemel, W. (2016). Increasing transparency through a multiverse analysis. Perspectives on Psychological Science, 11(5), 702–712. 10.1177/1745691616658637, 27694465

[bib150] Szewczyk, J. M., & Federmeier, K. D. (2022). Context-based facilitation of semantic access follows both logarithmic and linear functions of stimulus probability. Journal of Memory and Language, 123, 104311. 10.1016/j.jml.2021.104311, 36337731 PMC9631957

[bib151] Taylor, W. L. (1953). “Cloze procedure”: A new tool for measuring readability. Journalism Quarterly, 30(4), 415–433. 10.1177/107769905303000401

[bib185] Trott, S. (2024). Can large language models help augment English psycholinguistic datasets? Behavior Research Methods, 56(6), 6082–6100. 10.3758/s13428-024-02337-z, 38261264 PMC11335796

[bib154] Trott, S. (2025). Toward a theory of generalizability in LLM mechanistic interpretability research. arXiv. 10.48550/arXiv.2509.22831

[bib155] Trott, S., & Bergen, B. (2021). RAW-C: Relatedness of ambiguous words in context (A new lexical resource for English). In Proceedings of the 59th annual meeting of the association for computational linguistics and the 11th international joint conference on natural language processing (Volume 1: Long Papers) (pp. 7077–7087). Association for Computational Linguistics. 10.18653/v1/2021.acl-long.550

[bib156] Trott, S., & Bergen, B. (2023). Word meaning is both categorical and continuous. Psychological Review, 130(5), 1239–1261. 10.1037/rev0000420, 36892900

[bib157] Trott, S., Jones, C., Chang, T., Michaelov, J., & Bergen, B. (2023). Do large language models know what humans know? Cognitive Science, 47(7), e13309. 10.1111/cogs.13309, 37401923

[bib158] Trott, S., Taylor, S. M., Jones, C. R., Michaelov, J. A., & Rivière, P. D. (2026). Language statistics and false belief reasoning: Evidence from 41 open-weight LMS. Proceedings of the 64th Annual Meeting of the Association for Computational Linguistics (Volume 1: Long Papers) (pp. 39816–39833). Association for Computational Linguistics. 10.18653/v1/2026.acl-long.1849

[bib159] Trott, S., Walker, D. E., Taylor, S. M., & Coulson, S. (2025). Turing jest: Distributional semantics and one-line jokes. Cognitive Science, 49(5), e70066. 10.1111/cogs.70066, 40396401 PMC12093450

[bib160] van Berkum, J. J., Hagoort, P., & Brown, C. M. (1999). Semantic integration in sentences and discourse: Evidence from the N400. Journal of Cognitive Neuroscience, 11(6), 657–671. 10.1162/089892999563724, 10601747

[bib161] Van Petten, C. (2014). Examining the N400 semantic context effect item-by-item: Relationship to corpus-based measures of word co-occurrence. International Journal of Psychophysiology, 94(3), 407–419. 10.1016/j.ijpsycho.2014.10.012, 25448377

[bib162] Van Petten, C., & Luka, B. J. (2012). Prediction during language comprehension: Benefits, costs, and ERP components. International Journal of Psychophysiology, 83(2), 176–190. 10.1016/j.ijpsycho.2011.09.015, 22019481

[bib163] van Schijndel, M., & Linzen, T. (2021). Single-stage prediction models do not explain the magnitude of syntactic disambiguation difficulty. Cognitive Science, 45(6), e12988. 10.1111/cogs.12988, 34170031

[bib164] Vasishth, S., Nicenboim, B., Beckman, M. E., Li, F., & Kong, E. J. (2018). Bayesian data analysis in the phonetic sciences: A tutorial introduction. Journal of Phonetics, 71, 147–161. 10.1016/j.wocn.2018.07.008, 30197458 PMC6124675

[bib165] Vaswani, A., Shazeer, N., Parmar, N., Uszkoreit, J., Jones, L., Gomez, A. N., & Polosukhin, I. (2017). Attention is all you need. Advances in Neural Information Processing Systems, 30.

[bib166] Vinaya, H., Trott, S., Pecher, D., Zeelenberg, R., & Coulson, S. (2024). Context-dependent and dynamic effects of distributional and sensorimotor distance measures on EEG. In Proceedings of the Annual Meeting of the Cognitive Science Society (Vol. 46).

[bib167] Vinaya, H., Trott, S., Pecher, D., Zeelenberg, R., & Coulson, S. (2025). Words and worlds both: Dynamic effects of distributional and sensorimotor information in semantic processing. Open Mind: Discoveries in Cognitive Science, 9, 2149–2174. 10.1162/OPMI.a.316, 41497522 PMC12768550

[bib168] Wang, A., Singh, A., Michael, J., Hill, F., Levy, O., & Bowman, S. R. (2018). GLUE: A multi-task benchmark and analysis platform for natural language understanding. arXiv. 10.48550/arXiv.1804.07461

[bib169] Wang, K., Variengien, A., Conmy, A., Shlegeris, B., & Steinhardt, J. (2022). Interpretability in the wild: A circuit for indirect object identification in GPT-2 small. arXiv. 10.48550/arXiv.2211.00593

[bib170] Warstadt, A., Mueller, A., Choshen, L., Wilcox, E., Zhuang, C., Ciro, J., Mosquera, R., Paranjape, B., Williams, A., Linzen, T., & Cotterell, R. (2025). Findings of the BabyLM challenge: Sample-efficient pretraining on developmentally plausible corpora. arXiv. 10.48550/arXiv.2504.08165

[bib171] Wilcox, E. G., Gauthier, J., Hu, J., Qian, P., & Levy, R. (2020). On the predictive power of neural language models for human real-time comprehension behavior. arXiv. 10.48550/arXiv.2006.01912

[bib172] Wilcox, E. G., Meister, C. I., Cotterell, R., & Pimentel, T. (2023). Language model quality correlates with psychometric predictive power in multiple languages. In Proceedings of the 2023 conference on empirical methods in natural language processing (pp. 7503–7511). Association for Computational Linguistics. 10.18653/v1/2023.emnlp-main.466

[bib173] Winter, B. (2019). Statistics for linguists: An introduction using R. Routledge. 10.4324/9781315165547

[bib174] Yao, W., Yang, W., Wang, Z., Lin, Y., & Liu, Y. (2025). Understanding the capabilities and limitations of weak-to-strong generalization. In Scaling self-improving foundation Models without human supervision.

[bib175] Ziems, C., Held, W., Shaikh, O., Chen, J., Zhang, Z., & Yang, D. (2024). Can large language models transform computational social science? Computational Linguistics, 50(1), 237–291. 10.1162/coli_a_00502

